# Intraneuronal aggregation of the β-CTF fragment of APP (C99) induces Aβ-independent lysosomal-autophagic pathology

**DOI:** 10.1007/s00401-016-1577-6

**Published:** 2016-04-30

**Authors:** Inger Lauritzen, Raphaëlle Pardossi-Piquard, Alexandre Bourgeois, Sophie Pagnotta, Maria-Grazia Biferi, Martine Barkats, Pascale Lacor, William Klein, Charlotte Bauer, Frederic Checler

**Affiliations:** 1 Université de Nice-Sophia-Antipolis, Institut de Pharmacologie Moléculaire et Cellulaire, CNRS-UMR7275, Team Labelised Fondation pour la Recherche Médicale et Laboratoire d’excellence Distalz, Sophia-Antipolis, France; 2 CCMA-Université de Nice-Sophia-Antipolis, Nice, France; 3 Institut-Myologie, Paris, France; 4 Department of Neurobiology, Northwestern University, Evanston, IL USA

**Keywords:** Alzheimer, C99, Lysosomes, Autophagy, Aggregation, Triple-transgenic mouse, γ-Secretase inhibition

## Abstract

Endosomal-autophagic-lysosomal (EAL) dysfunction is an early and prominent neuropathological feature of Alzheimers’s disease, yet the exact molecular mechanisms contributing to this pathology remain undefined. By combined biochemical, immunohistochemical and ultrastructural approaches, we demonstrate a link between EAL pathology and the intraneuronal accumulation of the β-secretase-derived βAPP fragment (C99) in two in vivo models, 3xTgAD mice and adeno-associated viral-mediated C99-infected mice. We present a pathological loop in which the accumulation of C99 is both the effect and causality of impaired lysosomal-autophagic function. The deleterious effect of C99 was found to be linked to its aggregation within EAL-vesicle membranes leading to disrupted lysosomal proteolysis and autophagic impairment. This effect was Aβ independent and was even exacerbated when γ-secretase was pharmacologically inhibited. No effect was observed in inhibitor-treated wild-type animals suggesting that lysosomal dysfunction was indeed directly linked to C99 accumulation. In some brain areas, strong C99 expression also led to inflammatory responses and synaptic dysfunction. Taken together, this work demonstrates a toxic effect of C99 which could underlie some of the early-stage anatomical hallmarks of Alzheimer’s disease pathology. Our work also proposes molecular mechanisms likely explaining some of the unfavorable side-effects associated with γ-secretase inhibitor-directed therapies.

**Electronic supplementary material:**

The online version of this article (doi:10.1007/s00401-016-1577-6) contains supplementary material, which is available to authorized users.

## Introduction

Alzheimer’s disease (AD) is the most prevalent neurodegenerative disease and cause of dementia in elderly. Histopathologically, it is characterized by the deposition of extracellular amyloid plaques and intraneuronal neurofibrillary tangles, as well as synaptic pathology and neurodegeneration [7]. Growing evidence supports that these alterations are preceded and may be even caused by a defective endolysosomal/autophagic degradation [33, 36, 39, 43, 50]. Indeed, endolysosomal/autophagic anomalies are well-recognized early neuropathological features of AD, marked by enlarged endosomes, lysosomal alterations and progressive accumulation of autophagic vacuoles (AVs) [33–35, 39, 43, 50]. It is also well established that endosomal-autophagic-lysosomal (EAL) compartments constitute the main sites of proteolytic processing of the amyloid-β precursor protein βAPP [43] and that amyloidogenic species can accumulate inside organelles of the EAL machinery [12, 20, 24, 26, 62]. Moreover, the accumulation of the aggregate-prone Aβ42 within EAL organelles was found to disturb normal EAL function, suggesting that it could be both a consequence and a cause of endolysosomal-autophagic dysfunction [43].

EAL dysfunction has been extensively described in transgenic mice displaying AD-like anatomo-pathology [11, 13, 47, 51, 55, 61] including in the widely used 3xTgAD mouse (APP_Swe_, Tau_P301L_, PS1_M146V_) [10, 11, 37]. It is also well known that this mouse model displays an early accumulating and particularly high intraneuronal amyloid-like immunostaining within the subiculum of the hippocampus, which was firstly accounted for Aβ [37]. Yet, we recently demonstrated that this staining corresponded to the β-secretase-derived fragment, C99 (βCTF) rather than to Aβ, which was only detected at low levels and at late stages in these mice [24]. We found that C99 accumulated in enlarged cathepsin B-positive structures suggesting a link between C99 and EAL pathology [24]. In this work, we establish that C99 accumulation is the consequence of impaired lysosomal-autophagic degradation but that C99, in turn, also contributes to lysosomal dysfunction. Interestingly, these effects of C99 were observed in the 3xTgAD mouse, in which C99 is generated by proteolytically APP processing, but also in a direct transgenic C99-expressing mouse. C99 fragments were found to aggregate within membranes of EAL vesicles, proposing that C99 induces autophagic-lysosomal dysfunction by interfering with EAL membrane integrity. The pharmacological inhibition of γ-secretase increased the levels of EAL-associated C99 and exacerbated pathology, clearly demonstrating a C99 dose-dependent but Aβ-independent effect. Overall, our work demonstrates a detrimental loop in which aggregated C99 contributes to anatomical hallmarks reminiscent of those occurring early in Alzheimer’s disease.

## Materials and methods

### Viral constructions

Virus production was performed following a protocol previously described [4]. Briefly, HEK293 cells were transfected with the adenovirus helper plasmid (pXX6), the AAV packaging plasmid (rAAV2-rh10), and the AAV10 plasmid empty vector or encoding either human C99 or GFP under control of the synapsin-1 promoter (AAV-empty, AAV-synC99 and AAV-synGFP). Viruses were produced, purified and vector titers were determined by real-time PCR and expressed as viral genomes per ml (vg/ml) [4].

### Animals and in vivo drug treatment

3xTgAD (harboring PS1_M146V_, βAPP_swe_, and Tau_P301L_ transgenes) and non-transgenic (nonTg) mice [37] were generated from breeding pairs provided by Dr. LaFerla (Irvine, USA). 2xTgAD (PS1wt, βAPP_swe_ and Tau_P301L_) were produced by crossing 3xTgAD with nonTg mice, as described [38]. For AAV-mediated in vivo delivery, 1-day-old C57BL6 mice (Janvier Labs., France) were injected with 4 µl of AAV virus (5.5 × 10^12^ vg/ml (viral genomes per ml)) into the left lateral ventricle, as described [21] and mice were analyzed at 2 months post-AAV delivery. 5-month-old nonTg and 3xTgAD males or 2-month-old AAV-infected mice (males and females) were treated daily for 12 or 30 days with the γ-secretase inhibitor ELND006, referred to as D6 hereafter (30 mg/kg, Elan Pharmaceuticals, San Francisco, USA) [9, 45] or with vehicle alone (methylcellulose/polysorbate 80, Sigma) via oral gavage, as described [24]. All experimental procedures were in accordance with the European Communities Council Directive of 24 November 1986 (86/609/EEC) and local French legislation.

### Immunohistochemical analysis

Animals were deeply anesthetized with a lethal dose of pentobarbital and perfused transcardially with cold phosphate-buffered saline (PBS). Brains were fixed in 4 % Paraformaldehyde/PBS then embedded in paraffin and sliced (8 μm) or cut on a vibratome (50 μm). For FCA18, 82E1, NU1, 4G8 and 6E10 staining, sections were treated with formic acid (90 % or 50 %/5 min for paraffin and vibratome sections, respectively). Sections were then incubated at 4 °C overnight with primary antibodies (see supplementary table) followed by Alexa Fluor-conjugated antibodies (Molecular Probes, 1:1000). Cathepsin B labeling was amplified using the Vectorstain ABC kit (Vector) and streptavidin-Alexa594 (Molecular Probes, 1:1000). Nuclei were stained with DAPI (Roche, 1:20,000) and fluorescence was visualized using a confocal microscope (Fluoview10, Olympus). For DAB development, sections were incubated with HRP-conjugated secondary antibodies (Jackson ImmunoResearch, 1/1000) followed by DAB substrate (DAB impact, Vector). Neuronal nuclei were then stained with cresyl violet. For quantitative analysis of lamp1 staining in AAV-empty and AAV-C99 brain slices, images were thresholded, converted to mask and analyzed using Particle Analysis ImageJ plugin.

### Cell culture and experimental treatments

The human neuroblastoma cell line SH-SY5Y, naive or stably expressing APPswe or pcDNA3 (mock), previously described [40] was exposed to the following drugs for 16–20 h: D6 (50 nM to 5 μM, Elan Pharmaceuticals, San Francisco) in vehicle (methylcellulose/polysorbate 80, Sigma), DAPT (5 μM in DMSO, Sigma), leupeptin (10 μM in H_2_O, Sigma), PADK (5 μM in DMSO, Bachem), NH_4_Cl (10 mM in H_2_O, Sigma), pepstatin A (10 μM in EtOH, Enzo Life sciences), smer28 (50 μM in DMSO, Sigma) and bafilomycin A1 (50 nM in H20, Sigma). Some cells were transfected with GFP-LC3 (Addgene) using Lipofectamine 2000 according to standard protocols. Other cells were infected with lentiviruses (LV) encoding mouse cathepsin B (LV-mCatB-FUGW2) or control (LV-FUGW2) [32] and polyclonal cell lines stably expressing mCatB (naive SH-SY5Y or APPswe-SH-SY5Y cells) were obtained. For immunocytochemical experiments of C99 expression, COS-7 cells were transfected with C99 using Lipofectamine 2000.

### Immunocytochemistry

Cells were immunostained with α-APPct (1:5000) and α-lamp-1 (E-5, 1:100) or α-cis-golgi (GM130, Cell signaling, 1:400) followed by Alexa Fluor-488 or Alexa Fluor-594 conjugated antibodies (1:1000, Molecular Probes). Nuclei were stained with DAPI (1:20,000, Molecular probes). Other cells were incubated 30 min with Lysotracker-Red DND-99 (1:20,000, Invitrogen) at 37 °C, fixed with PFA 4 % and stained with DAPI. Images were acquired using an inverted confocal microscope (Fluoview10, Olympus, France).

### In vitro degradation assay

Recombinant C99 (C100-flag), previously described [49], was incubated in the absence or presence of purified cathepsin B from human placenta (5, 10 or 50 ng, Sigma) in 100 mM sodium acetate pH 5 buffer containing 1 mM EDTA and 8 mM cysteine at 37 °C during 60 min, then analyzed on Tris-Tricine 16 % acrylamide gels as described below. C100flag was revealed using an anti-flag antibody (M2, 1:5000, Sigma).

### Electron microscopy

Cells were fixed 20 min with 2.5 % glutaraldehyde/phosphate buffer 0.1 N (pH 7.4). Mice were anesthetized with a lethal dose of pentobarbital and perfused transcardially with ice cold physiological saline followed by 2 % glutaraldehyde/cacodylate buffer 0.1 M (pH 7.4). The brain was sliced (200 μm) on a vibratome and 2 mm cubes from the subiculum were microdissected under binoculars and post-fixed in osmium tetroxide (1 % in cacodylate buffer 0.1 M). The tissue was embedded in Epon resin (EMS) and 80 nm ultrathin sections were contrasted with uranyl acetate and lead citrate and visualized using a JEM 1400 electron microscope operating at 100 kV equipped with a Morada SIS camera.

### Preparation of brain fractions

Dissected hippocampi of vehicle or D6-treated 3xTgAD or nonTg mice, or hemispheres from AAV-injected mice, were homogenized in RIPA buffer (Tris 50 mM; pH 7.4 containing NaCl (150 mM), EDTA (1 mM), Triton X100 (1 %), deoxycholate (0.5 %), SDS (0.1 %) and protease inhibitor cocktail (Sigma) and soluble and insoluble fractions were prepared as described [24]. Synaptosomal and microsomal enriched fractions were prepared from hippocampi of vehicle or D6-treated 3xTgAD mice. Tissue was homogenized in 0.32 M sucrose containing protease inhibitor cocktail (Sigma), centrifuged twice at 850×*g*/5 min and the collected supernatants were centrifuged twice at 12,000×*g*/20 min. The obtained pellet (“synaptomal fraction”) was resuspended in Hepes 4 mM/EDTA. The supernatant was centrifuged at 20,000×*g*/1 h to obtain the “microsomal fraction”.

### SDS/PAGE and western blot analyses

Proteins were separated on 16 % Tris-Tricine gels (for APP-CTFs, cathepsin B and LC3) or 10 % Tris–glycine gels (for βAPP p62 and actin) and transferred to nitrocellulose membranes. After probing with primary antibodies (see supplementary table), immunological complexes were revealed with HRP-conjugated antibodies (Jackson ImmunoResearch, 1/10,000) followed by electrochemiluminescence (Supersignal West Pico/West Maximum Sensitivity chemiluminescence substrate, Thermo Scientific, France). Peak height of signal intensities from protein bands were quantified with ImageJ software.

### Quantification of human Aβ

Aβ40 and Aβ42 levels were quantitatively detected in soluble and insoluble fractions (see above) of mice hippocampi (for 3xTgAD mice) or hemispheres (for AAV-synC99 mice) using sandwich ELISA kits detecting human Aβ40 and Aβ42, respectively (Biosource, Invitrogen, France). Absorbance was read at 450 nm using a spectrophotometer.

### In vitro cathepsin B activity assay

Cells were lysed mechanically in homogenization buffer (250 mM sucrose, 1 mM EDTA, 5 mM Hepes pH 7.4). The cell suspension was centrifuged at 850×*g*/5 min and the supernatant was centrifuged at 20,000×*g*/90 min. The pellet (membrane enriched fraction) was resuspended in Tris–HCl (10 mM, pH 7.5). To monitor cathepsin B (CatB) activity, 60 µg of protein extracts were incubated in acetate buffer (25 mM, pH 5.5, 100 µl and 8 mM l-cysteine HCl) containing CatB substrate (CBZ-Arg-Arg-7-Amido-4-methylcoumarin, 100 µM, Sigma) in the absence or presence of leupeptin (10 µM, Sigma). Specific CatB activity corresponds to the leupeptin-sensitive fluorescence recorded at 320 nm (excitation) and 420 nm (emission) using a fluorescence plate reader (FLUOstar Omega, BMG Labtech, France). CatB activity was calculated as the slope in the linear range corresponding to the initial 30 min.

### Electrophysiological recordings

LTP measurements were performed by E-Phy-Science, Sophia Antipolis, France. 2-month-old AAV-empty, vehicle or D6-treated AAV-C99 females, were anesthetized with isoflurane and decapitated. Stimulations were delivered in CA1 fibers in the alveus and field excitatory postsynaptic potential (f-EPSP) were recorded from the subicular pyramidal neurons in the middle portion of the subiculum. The LTP-induction protocol consisted of four trains of 100 stimuli at 100 Hz repeated every 20 s, as described [41].

### Statistical analysis

Statistical analysis was performed with PRISM software (Graph-Pad Software, San Diego, CA, USA) by using a non-parametric two-tailed Mann–Whitney *U* test for pairwise comparisons or the two-way ANOVA test followed by either the Tukey’s post hoc test for multiple comparisons or the Dunnett post hoc test when comparison to controls. All data are expressed as the mean ± SEM. Differences were considered significant at *p < 0.05, **p < 0.01, ***p < 0.001.

## Results

### 3xTgAD and 2xTgAD mice display early and progressive C99 accumulation within enlarged cathepsin B and lamp1 positive structures

**Fig. 1:**
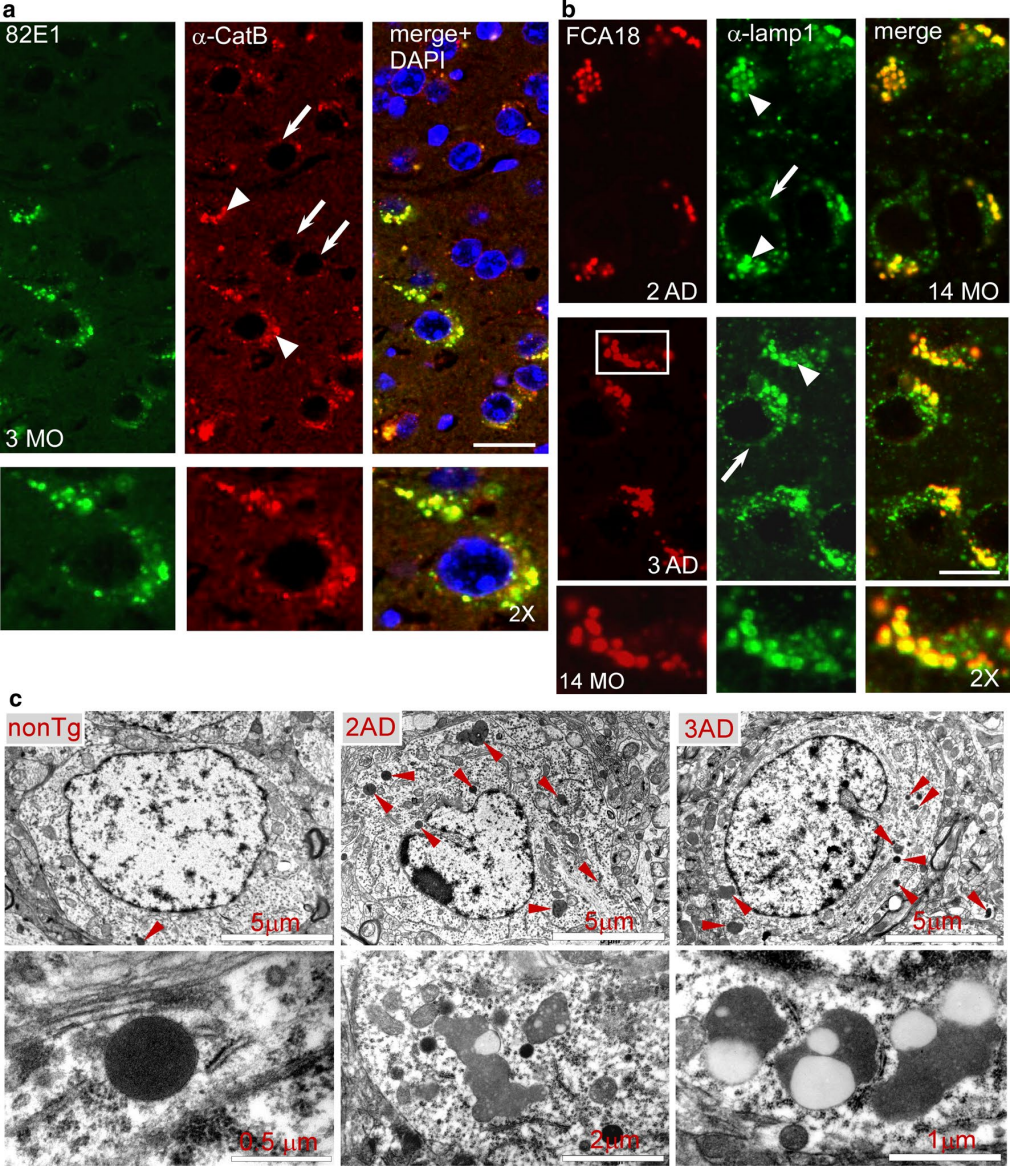
C99 accumulates in enlarged cathepsin B- and lamp1-positive structures. **a** Co-immunohistochemical staining of C99 with 82E1 (*green*) and α-cathepsin B (*red*) in 3-month-old 3xTgAD mice. Note the co-localization (merged image with DAPI staining) of the two labelings and the presence of enlarged (*arrowheads*) cathepsin B structures in 82E1-positive cells as compared to normal-sized cathepsin B structures (*arrows*) in 82E1-negative cells. *Scale bar* 20 μm. **b** Co-immunohistochemical staining with FCA18 (*red*) and α-lamp1 (*green*) shows the localization of the FCA18-associated staining within enlarged lamp1 structures in both 14-month-old 2xTgAD (2AD) and 3xTgAD (3AD) mice. *Scale bar* 10 μm. **c** Electron microphotographs were taken from the subiculum of 14-month-old non-transgenic (nonTg), 2AD or 3AD mice. *Upper panels* correspond to low-magnification images of representative neurons from nonTg, 2AD or 3xTgAD mice (*scale bar* 5 μm). The *lower images* illustrate examples of lysosomal-dense bodies (<0.2 μm, *left image*), or of bigger membrane-limited structures of homogenous density corresponding to autolysosomes (>0.5 μm, *middle panels*) and giant autolysosomes (>1.5 μm, *middle* and *right panels*)

We previously demonstrated that the β-secretase-derived APP C-terminal stub C99 corresponded to the early appearing and accumulating intracellular label in the subiculum of 3xTgAD mice [24]. This label could be detected with N-terminal directed antibodies including the β-secretase-mediated cleavage-specific antibodies FCA18 [3] and 82E1 [19] known to recognize both Aβ and C99. A similar accumulating intraneuronal FCA18-immunostaining was observed in double transgenic (2xTgAD) mice harboring APP_swe_ and Tau_P301L_ but wild-type presenilin1 [24] (Suppl. Figure 1a). Interestingly, in the two strains this intracellular staining (Suppl. Figure 1a) and C99 levels (Suppl. Figure 1c, d) increased similarly with age, while only 3xTgAD mice produced extracellular plaques at late ages that were stained with both FCA18 (Suppl. Figure 1a) and anti-Aβ42 (α-Aβ42) (Suppl. Figure 1b). The detection of very few extracellular Aβ plaques in situ in aged 2xTgAD mice was corroborated by the absence or very low detectable levels of both Aβ40 and Aβ42 measured by ELISA (Suppl. Figure 1e). Overall, these data indicated that C99 accumulation did not correlate with Aβ load and therefore could not be accounted for altered γ-secretase processing alone. In situ immunolabeling with 82E1 revealed that C99 accumulated in intraneuronal organelles positive for the lysosomal enzyme cathepsin B (Fig. 1a) and suggested a link between C99 accumulation and defective lysosomal clearance. In young 3xTgAD mice (3 months of age), the majority of the neurons displayed numerous small-sized cathepsin B puncta (Fig. 1a, arrows), but some neurons also presented enlarged cathepsin B structures which were positive for C99 (Fig. 1a, arrowheads). Similar results were obtained with an antibody directed against the endosomal-lysosomal membrane-associated protein lamp1, and the number and size of these structures were high in both 14-month-old 2AD and 3AD mice (Fig. 1b). The large size of these structures proposed that many of them corresponded to endo- or autolysosomes rather than to lysosomes and would support impaired lysosomal-autophagic-mediated C99 degradation in both 2xTgAD and 3xTgAD mice. In agreement, electron microscopy revealed an increased number of dense autophagic vesicles in brains from both of these mice, as compared to non-transgenic mice (Fig. 1c, red arrowheads). These autophagic vacuoles corresponded to both small and very dark autolysosomes (<500 nm, lower left image) and giant autolysosomes (>1.5 µm, examples in lower middle and right images) containing various compositions of densely packed undigested material and lipid droplets (clear round structures).

### Cellular analysis shows that APP-CTFs are cleared through the autophagic-lysosomal degradation pathway

**Fig. 2:**
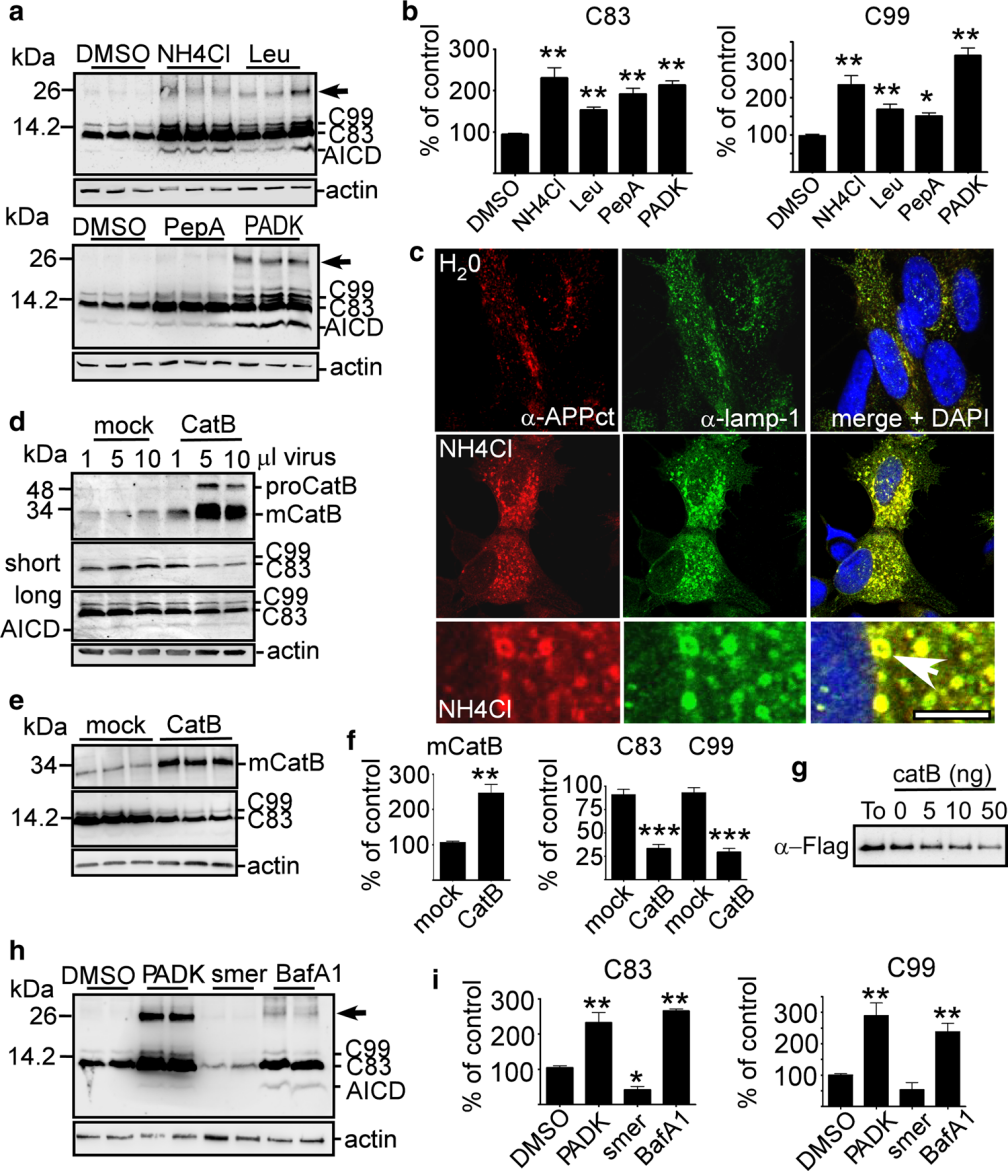
In vitro analysis shows that APP-CTFs are degraded by cathepsins through autophagy. **a**, **b** SH-APPswe cells were treated with NH_4_Cl, leupeptin (Leu), pepstatin A (PepA) or PADK and analyzed by western blot using α-APPct. *Arrows* correspond to a non-identified 25 kDa APP-CTF fragment. *Bars* in **b** correspond to quantification of C99 and C83 immunoreactivities obtained in **a** and expressed relative to expressions measured in DMSO-treated cells normalized to actin. Data are represented as mean ± SEM, as determined by ANOVA one-way Dunnett post hoc test, **p < 0.01 and *p < 0.05 indicate significant differences relative to control cells (*n* = 10–16, from four independent experiments). **c** Immunocytochemical analysis using α-APPct and α-lamp1 on H_2_O or NH_4_Cl treated SH-APPswe cells. Note the labeling of α-APPct in enlarged α-lamp1 vesicles in merged images (*arrow*). **d** SH-APPswe cells were infected with lenti-cathepsin B (CatB) or empty vector (mock) virus at different concentrations (1, 5 or 10 μl of a stock corresponding to 3.75 × 10^8^ TU/ml) and CatB and APP-CTF levels were analyzed after 48 h by western blot. α-cathepsin B revealed immature (proCatB) and mature (mCatB) cathepsin B. **e**, **f** CatB and APP-CTF levels were analyzed by western blot in CatB stable cell lines after six passages. *Bars* in **f** represent the quantification of mCatB and C83 and C99 immunoreactivities obtained in **e**, each expressed as respective expressions measured in mock cells. Data are represented as mean ± SEM, Mann–Whitney, ***p < 0.001 and **p < 0.01 (*n* = 7 from three independent experiments). **g** Recombinant C100-flag was incubated in the absence or presence of increasing concentrations of purified CatB at 37 °C during 0 (To, 50 ng) or 60 min (5,10 or 50 ng) and C100 levels were detected by western blot using α-Flag antibody. **h**, **i** SH-APPswe cells were treated with smer-28 (smer), PADK or bafilomycin A1 (BafA1), and APP-CTF levels were analyzed by western blot using α-APPct. *Arrow* corresponds to a non-identified 25 kDa APP-CTF fragment. *Bars* in **i** correspond to quantification of C83 and C99 immunoreactivities obtained in **h** and expressed as the percentage of the expressions in DMSO-treated cells normalized to actin. Data are represented as mean ± SEM, as determined by ANOVA one-way Dunnett post hoc test, **p < 0.01 and *p < 0.05 indicate significant differences relative to control cells *n* = 8, from three independent experiments

The above data suggested a role of lysosomal-autophagic degradation in C99 accumulation and would agree with earlier data proposing a major contribution of this degradation pathway in APP-CTF clearance [1, 8, 54, 57]. To confirm the role of lysosomal enzymes in the fate of C99, we used SH-SY5Y neuroblastoma overexpressing APPswe (known to potentiate β-secretase-mediated C99 and Aβ production [29] and harbored by the βAPP transgene in both 2xTgAD and 3xTgAD mice [37]) (SH-APPswe). Clearly, treatments of SH-APPswe with either the lysosomotrophic weak base NH_4_Cl or specific lysosomal protease inhibitors, such as the cathepsin B inhibitors leupeptin and PADK or the cathepsin D inhibitor pepstatin A, all led to large increases in APP-CTFs, including the most abundantly expressed C83, C99 and AICD (Fig. 2a, b). These treatments also led to the accumulation of other higher molecular mass APP-CTFs, notably one about a size of 25 kDa (Fig. 2a, arrows), whose levels were very low in non-treated cells, showing its particularly high sensitivity to lysosomal degradation. Although, we did not determine the exact identity of this fragment, it could possibly correspond to the recently described ƞ-secretase-derived APP-CTF [2, 59]. Immunostaining showed that these APP-CTFs strongly accumulated in lamp1-positive structures (Fig. 2c) and similarly to lamp1, they localized at their membranes (Fig. 2c, arrow). The fact that they were more numerous and much larger in NH_4_Cl-treated cells than in control cells suggested that many of them could represent endo- or autolysosomes instead of simple lysosomes (Fig. 2c). If C99 load is genuinely increased by a defect of the enzymatic machinery, one should conversely expect to reduce C99 levels by enhancing lysosomal enzyme expression. Indeed, reduced APP-CTF levels were observed in both lentiviral-induced cathepsin B overexpressing cells (Fig. 2d) and in cells stably expressing this enzyme (Fig. 2e, f), as compared to mock-transfected cells. Furthermore, to demonstrate the ability of cathepsin B to catabolize C99 directly, we designed a bimolecular enzymatic assay in which various amounts of recombinant cathepsin B were incubated with recombinant C99 (C100-flag). This experiment confirmed the direct degradation of C99 by cathepsin B (Fig. 2g). Finally, we used a pharmacological approach to either activate or inhibit autophagy by using the small molecule enhancer of rapamycin, smer-28 [54] or the vacuolar-type H+ ATPase inhibitor, bafilomycin A1, respectively. As expected, APP-CTFs levels were reduced by smer-28 and enhanced by both bafilomycin A1 and the cathepsin B inhibitor PADK (Fig. 2h, i). All together, these findings highlighted a critical role of lysosomal-autophagic function in APP-CTF clearance and clearly supported our hypothesis connecting impaired lysosomal-autophagic degradation and C99 accumulation.

### In vivo treatment of young 3xTgAD mice with the γ-secretase inhibitor ELND006 triggers massive increases in EAL-associated APP-CTFs and leads to autophagic pathology

**Fig. 3:**
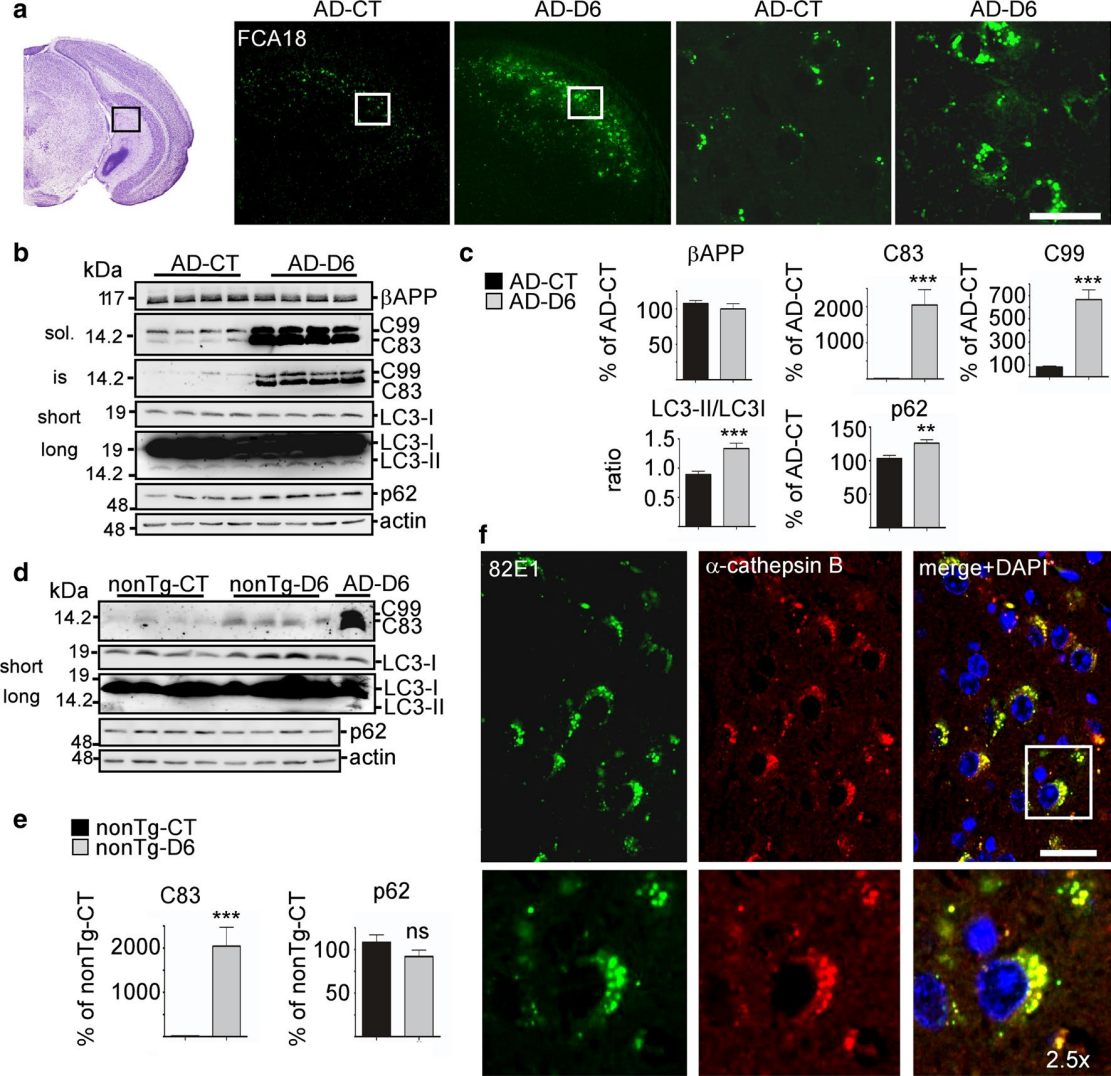
In vivo treatment of 3xTgAD mice with the γ-secretase inhibitor ELND006 (D6) leads to increased APP-CTFs localized to large cathepsin B-positive structures and to autophagic impairment. 5-month-old non-transgenic (nonTg) or 3xTgAD males were treated daily with D6 or vehicle by oral gavage during 1 month. **a** Immunostaining with FCA18 in the subiculum (see *insert*) of 3xTgAD vehicle- (AD-CT) or D6-treated (AD-D6) mice. *Scale bar* 125 and 20 μm, respectively. **b**, **c** RIPA-soluble (sol.) fractions from hippocampi of AD-CT or AD-D6 mice were analyzed for βAPP, APP-CTF, LC3-I, LC3-II and p62 expression by western blot. LC3-II was revealed after long time exposure of the same blot revealed for LC3-I. RIPA insoluble acid formic retrieved fractions (is) were analyzed for APP-CTF expression. *Bars* in **c**, are the mean ± SEM of 12 animals of each treatment and represent the quantification of βAPP, C83, C99 and p62 immunoreactivities expressed as the percentage measured in AD-CT mice normalized to actin, and the LC3-II to LC3-I ratio normalized to AD-CT. Statistical analysis was performed by Mann–Whitney and ***p < 0.001 and **p < 0.01 indicate significant differences relative to AD-CT. **d**, **e** RIPA-soluble fractions from hippocampi of non-transgenic vehicle- (nonTg-CT) or D6-treated (nonTg-D6) mice were analyzed for APP-CTF, LC3-I, LC3-II and p62 expression by western blot. *Right lane* corresponds to AD-D6 mice. *Bars* in **e** are the mean ± SEM of six animals of each treatment and represent the quantification of C83 and p62 immunoreactivities expressed as the percentage measured in nonTg-CT mice. Statistical analysis was performed by Mann–Whitney and ***p < 0.001 indicate significant differences relative to nonTg-CT. **f** Images correspond to co-immunohistochemical staining with 82E1 and α-cathepsin B in subiculum of AD-D6 mice. *Scale bar* 20 μm

We then questioned whether C99 per se could contribute to lysosomal-autophagic dysfunction. To this end, we took advantage of a pharmacological approach in which we used the γ-secretase inhibitor ELND006 (D6) found to induce enhanced C99 (and C99-derived C83) levels [24]. Young mice were treated daily for 1 month with D6 and analyzed for C99 expression by immunohistochemistry (Fig. 3a, f) or western blot (Fig. 3b, c) as well as for lysosomal function (Fig. 3b, c). As expected, FCA18-associated immunolabeling showed a remarkable increase in lysosomal-associated C99 staining in brains of D6-treated mice (AD-D6) as compared to those of vehicle-treated mice (AD-CT) (Fig. 3a). This pharmacological treatment also drastically enhanced the number of enlarged cathepsin B-positive structures in C99-containing neurons indicating concomitant C99 accumulation and exacerbated lysosomal pathology (Fig. 3f). These observations were confirmed by western blot analysis revealing significant increases in the autophagic marker LC3-II and the autophagic substrate p62/SQSTM1 (Fig. 3b, c), thus indicating autophagic impairment [6]. In wild-type (nonTg) mice, the treatment with the inhibitor also led to increased levels of C83, which however remained very low as compared to those observed in AD-D6 mice (right lane in the western blot) (Fig. 3d, e). In both vehicle and D6-treated nonTg mice, LC3-II was not detected and p62 levels were unchanged (Fig. 3d, e) indicating the absence of effect of D6 on autophagic function in these mice.

**Fig. 4:**
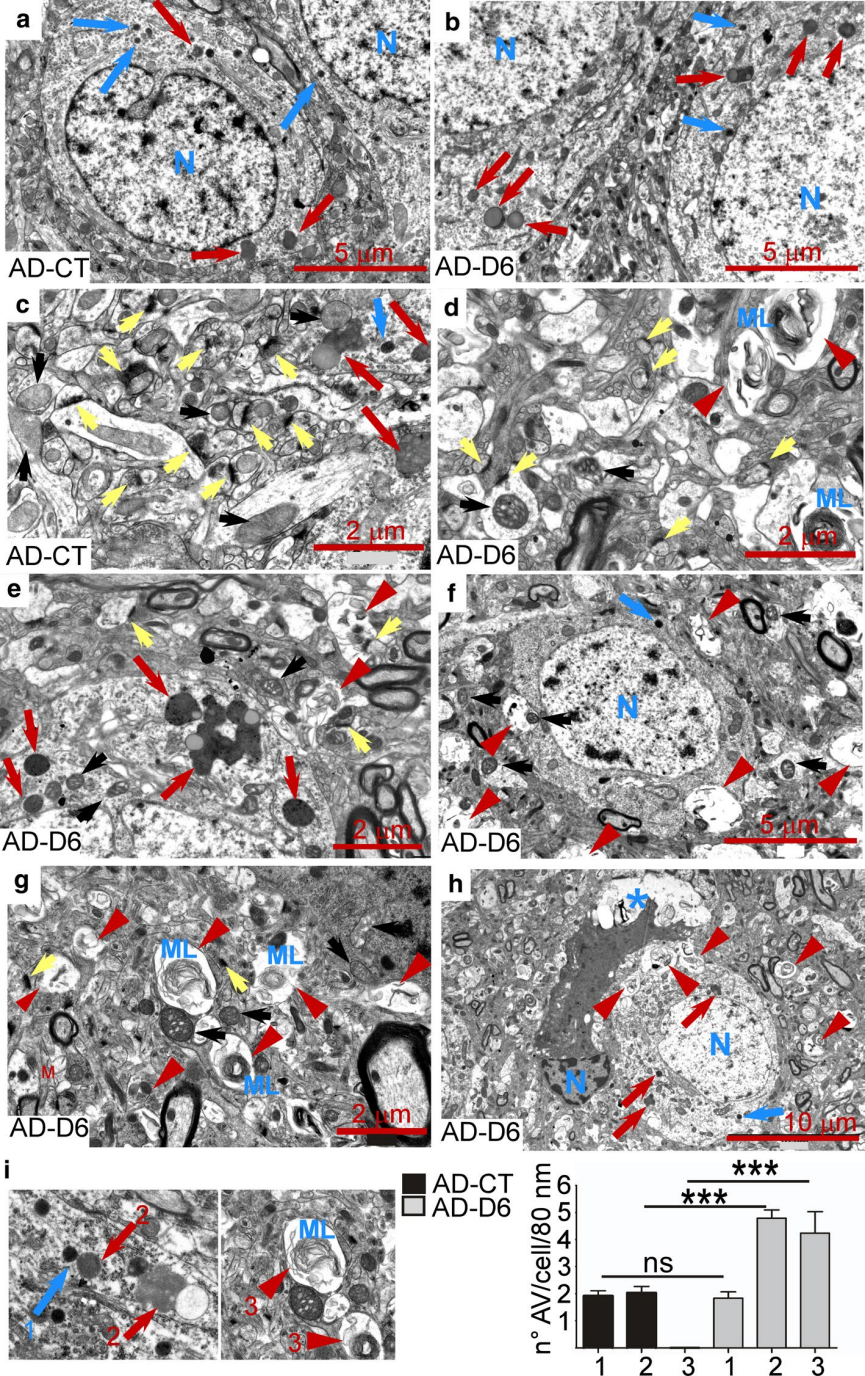
In vivo treatment of 3xTgAD mice with the γ-secretase inhibitor ELND006 (D6) leads to autophagic pathology and intraneuronal damage. Electron microphotographs of neuronal somas and neuropil from 5-month-old 3xTgAD males treated with D6 (AD-D6) (**b**, **d**, **e**, **h**) or vehicle (AD-CT) (**a**, **c**) during 1 month. Neuronal perikarya of AD-CT and AD-D6 mice contained dense lysosomal bodies (*blue arrows*) (**a**, **b**) and lipid-containing autophagic vacuoles (*red arrows*) (**a**), that are more abundant and enlarged in AD-D6 mice (*red arrows*) (**b**, **e**, **h**). The AD-D6 mice also displayed multiple large vesicles filled with heterogenous material (**d**–**h**—*red arrowheads*) and multilamellar bodies (**d**, **g**—*blue* ML).The neuropil of AD-CT mice presented many normal appearing synaptic contacts characterized by typical synaptic post-densities (PSDs, *yellow arrows* in **c**) and normal mitochondria (*black arrows*). In contrast, the neuropil in AD-D6 mice displayed very few normal appearing synaptic contacts (**d**—*yellow arrows*), many damaged mitochondria (*black arrows*, **f**, **g**) and electron-lucent areas (**h**—*blue stars*). *Scale bars* are 2 μm in **c**, **d**, **e**, **g**, 5 μm in **a**, **b**, **f**, and 10 μm in **h**. **i** Quantification of autophagic structures in slices from AD-CT (*black bars*) and AD-D6 (*grey bars*) mice. The autophagic structures (AVs) were classified in three (1–3) distinct groups, 1 corresponding to small dense AVs (*blue arrows*, less than 1 μm), 2 to a mix of larger sized and more or less lipid-containing AVs (*red arrows*) and 3 to large vesicles containing membranous material and multilamellar bodies (*red arrowheads*). *Bars* correspond to the average number of AVs per neuron (per cross section) and a count of 40–50 neurons per mouse (2 mice for each condition). Data are represented as mean ± SEM and statistical analysis was performed using the Mann–Whitney test and ***p < 0.001 indicate significant differences relative to AD-CT

In light of these observations, electron microscopy analysis (EM) was performed to confirm the above-described autophagic alterations. The subiculum of young AD vehicle-treated mice (AD-CT) displayed some small dense bodies (Fig. 4a, blue arrows) and various-sized dense lipid-containing autophagic vacuoles (Fig. 4a, red arrows) localized to perikarya, while age-matched vehicle-treated (nonTg-CT) (Suppl. Figure 2a) or D6-treated nonTg (nonTg-D6) neurons (Suppl. Figure 2b) presented very few of such structures. In D6-treated AD animals (AD-D6), EM revealed a strong autophagy-related pathology and intraneuronal damage (Fig. 4b, d–i and Suppl. Figure 2e–j). The number of small dense structures (<0.5 μm, blue arrows “1” in Fig. 4i) was identical in AD-CT and AD-D6 mice, but the number of larger sized autophagic vesicles, many containing lipids (>0.5 μm, red arrows “2” in Fig. 4i), was significantly increased in AD-D6 mice. However, the most striking feature of the autophagy-related pathology was the presence of multiple large vacuoles filled with heterogenous non-digested cargoes and multilamellar bodies (blue ML) (Fig. 4d, e–i and Suppl. Figure 2e–j—red arrowheads), (red arrowheads “3” in Fig. 4i), probably corresponding to immature autophagic vesicles [62]. Moreover, many AD-D6-treated neurons exhibited large electron-lucent areas of the cytoplasm localizing close to autophagic vesicles, suggesting a link between autophagic leakage and erosive destruction (Fig. 4h, and Suppl. Figure 2f, j—blue star). D6-treated neurons also presented a high number of damaged mitochondria (see examples in Fig. 4d, g and Suppl. Figure 2g, i—black arrows) and reduced synaptic contacts within the neuropil (Fig. 4d, yellow arrows), as compared to control mice (Fig. 4c—yellow arrows).

We next compared the effect of D6-treatment on APP-CTF accumulation in 2xTgAD and 3xTgAD mice. This treatment led to same increases in C99 (and C99-derived C83) (Suppl. Figure 3a, b) and in punctiform staining (Suppl. Figure 3d) in the two strains, whereas the effect of D6 on Aβ load was detected only in 3xTgAD mice displaying enough Aβ do detect a decrease (Suppl. Figure 3c). These data fully agreed with our proposal of a dual effect of D6 on APP-CTF accumulation, one linked to its canonical inhibitory effect of γ-secretase and one to its inhibitory effect on lysosomal degradation. In agreement with defective lysosomal degradation, both D6-treated 2xTgAD and 3xTgAD animals displayed enhanced, rather than lowered levels of the other γ-secretase cleavage-derived metabolite AICD (Suppl. Figure 3a, b) another lysosomal substrate (Fig. 2a) (see also [56]).

### The γ-secretase inhibitor ELND006 triggers lysosomal APP-CTF accumulation, reduced cathepsin B activity and autophagic impairment in SH-APPswe cells, but not in SH-mock cells

**Fig. 5:**
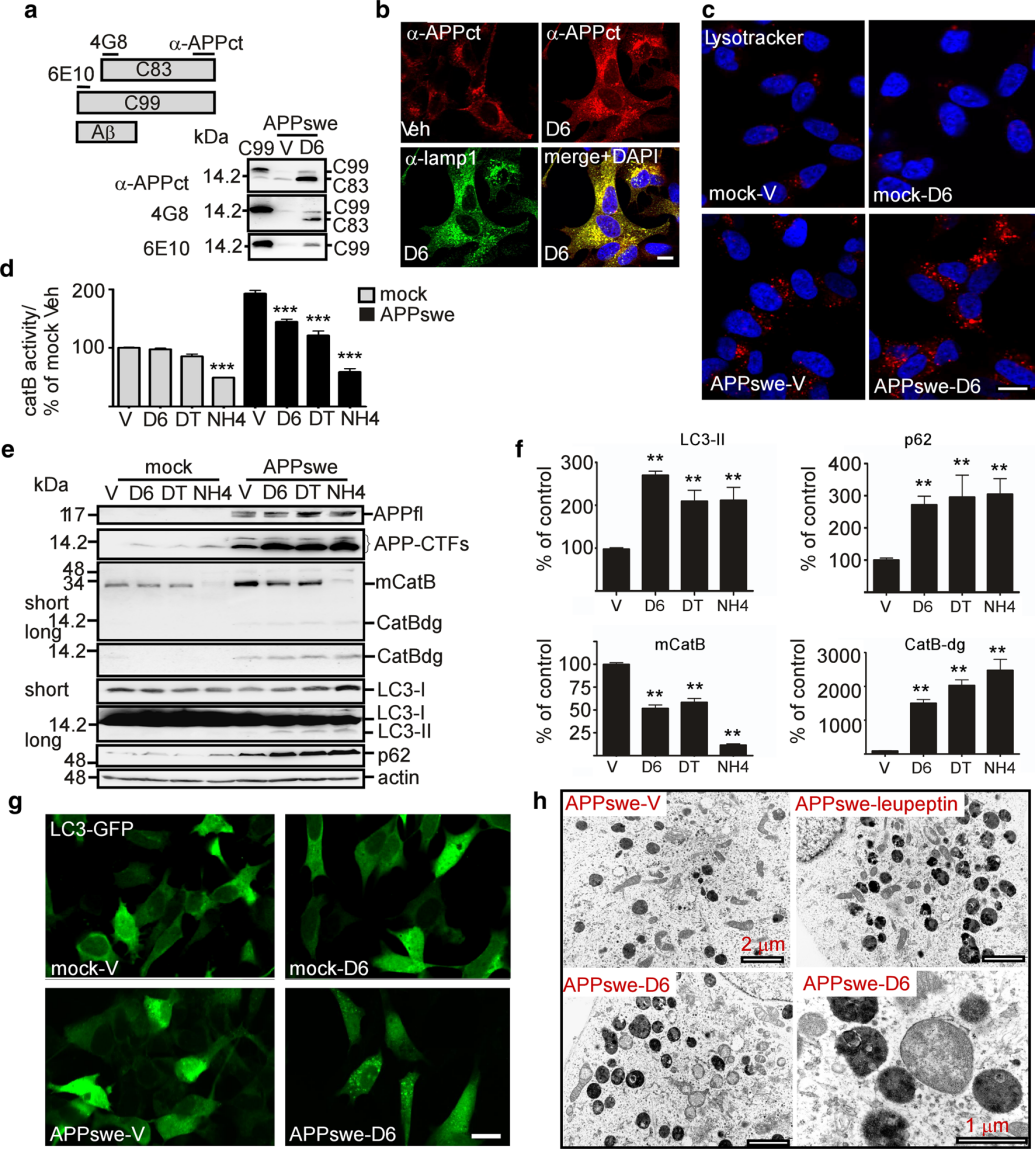
γ-Secretase inhibitor treatment triggers autophagic dysfunction in APPswe-expressing SHSY-5Y but not mock-transfected cells. **a** SH-APPswe cells were treated with ELND006 (D6) and analyzed for APP-CTF levels by western blot. APP-CTFs were detected using α-APPct, 4G8 or 6E10 (see diagram for epitope recognition) and compared to C99-flag expression in HEK transfected cells. **b** Co-immunocytochemical staining of vehicle (Veh) or D6-treated APPswe cells using α-APPct and α-lamp1 shows a high overlap of these labelings. *Scale bar* 2 μm. **c** Vehicle (V) or D6-treated SH-mock or SH-APPswe cells were stained with Lysotracker red and DAPI. *Scale bar* 5 μm. **d** Cathepsin B (CatB) activity was monitored in vitro from microsomal fractions of SH-APPswe cells treated with vehicle (V), D6, DAPT (DT) or NH_4_Cl (NH4). Data are represented as mean ± SEM, as determined by ANOVA one-way Dunnett post hoc test, ***p < 0.001 indicate significant differences relative to control cells (vehicle-treated cells), *n* = 8–14 from 2 independent experiments. **e**, **f** Stably CatB expressing mock or APPswe cells were treated with vehicle (V), D6, DAPT (DT) or NH_4_Cl and western blot analysis was used to determine the levels of βAPP and APP-CTFs, LC3-I, LC3-II, p62, CatB and actin. Besides the detection of the 30 kDa immunoreactivity corresponding to mature CatB, the CatB antibody revealed immunoreactivity at about 12 kDa most probably corresponding to a degradation product of this enzyme (CatB-dg.). **f** Quantification of immunoreactivities obtained in **e** for APPswe cells were normalized to actin and expressed as the percent of expression levels obtained in control treated cells. **g** SH-mock or SH-APPswe cells were transfected with LC3-GFP, treated with vehicle or D6 and was visualized by fluorescence microscopy. *Scale bar* 5 μm. **h** Electron microphotographs show representative cells from each condition. Scale bars are 1 or 2 μm, as indicated

To more precisely dissect the C99-mediated molecular mechanisms, we studied the effect of D6 treatment on lysosomal function in SH-APPswe cells. Firstly as expected, D6 triggered a drastic decrease in extracellular Aβ (not shown) and concomitant increase in C99 and C83 (Fig. 5a). Immunocytochemical analysis showed that these APP-CTFs were particularly abundant in lamp1-positive structures (Fig. 5b), which were enlarged and more numerous than those observed in control cells, similarly to cells treated with lysosomal inhibitors (Fig. 2c). These data were confirmed by Lysotracker-red, which labeled many and mostly large-sized puncta in D6-treated cells as compared to vehicle-treated cells (Fig. 5c). No increase in Lysotracker-red staining was seen in D6-treated SH-mock cells (i.e., cells only expressing endogenous levels of wild-type APP), suggesting a strong link between lysosomal dysfunction and APP-CTF accumulation. In addition, four independent lines of data supported our hypothesis, when we compared the effects of D6 and DAPT (another well-known γ-secretase inhibitor [58]) in SH-APPswe cells to SH-mock cells. Firstly, D6 and DAPT reduced in vitro cathepsin B activity (Fig. 5d) and lowered the levels of mature cathepsin B (Fig. 5e, f) in SH-APPswe but not in SH-mock cells (Fig. 5e), while NH_4_Cl strongly inhibited cathepsin B in both cell types (Fig. 5d–f). Moreover, in SH-APPswe cells both γ-secretase inhibitors led to the accumulation of a low-molecular weight (12 kDa) cathepsin B immunopositive product, probably corresponding to a degradation product of this enzyme (CatBdg) and supporting defective lysosomal clearance (Fig. 5e, f). Secondly, D6 and DAPT increased the levels of the autophagic markers LC3-II and p62 in only APPswe cells and the levels of these markers correlated with those of APP-CTFs (Fig. 5e, f). Thirdly, D6 treatment significantly increased LC3-positive vesicular profiles in SH-APPswe cells, whereas little if any LC3-GFP puncta were found in D6-treated SH-mock cells (Fig. 5g). Fourth, electron microscopy revealed the presence of large (0.5–2 μm) abnormal dense autolysosomal structures in D6-treated SH-APPswe cells, which structurally looked similar to those observed in leupeptin-treated cells (Fig. 5h).

### Adeno-associated viral vector (AAV)-mediated expression of C99 in wild-type mice leads to the accumulation of aggregated C99 in enlarged cathepsin B and lamp1-positive structures

**Fig. 6:**
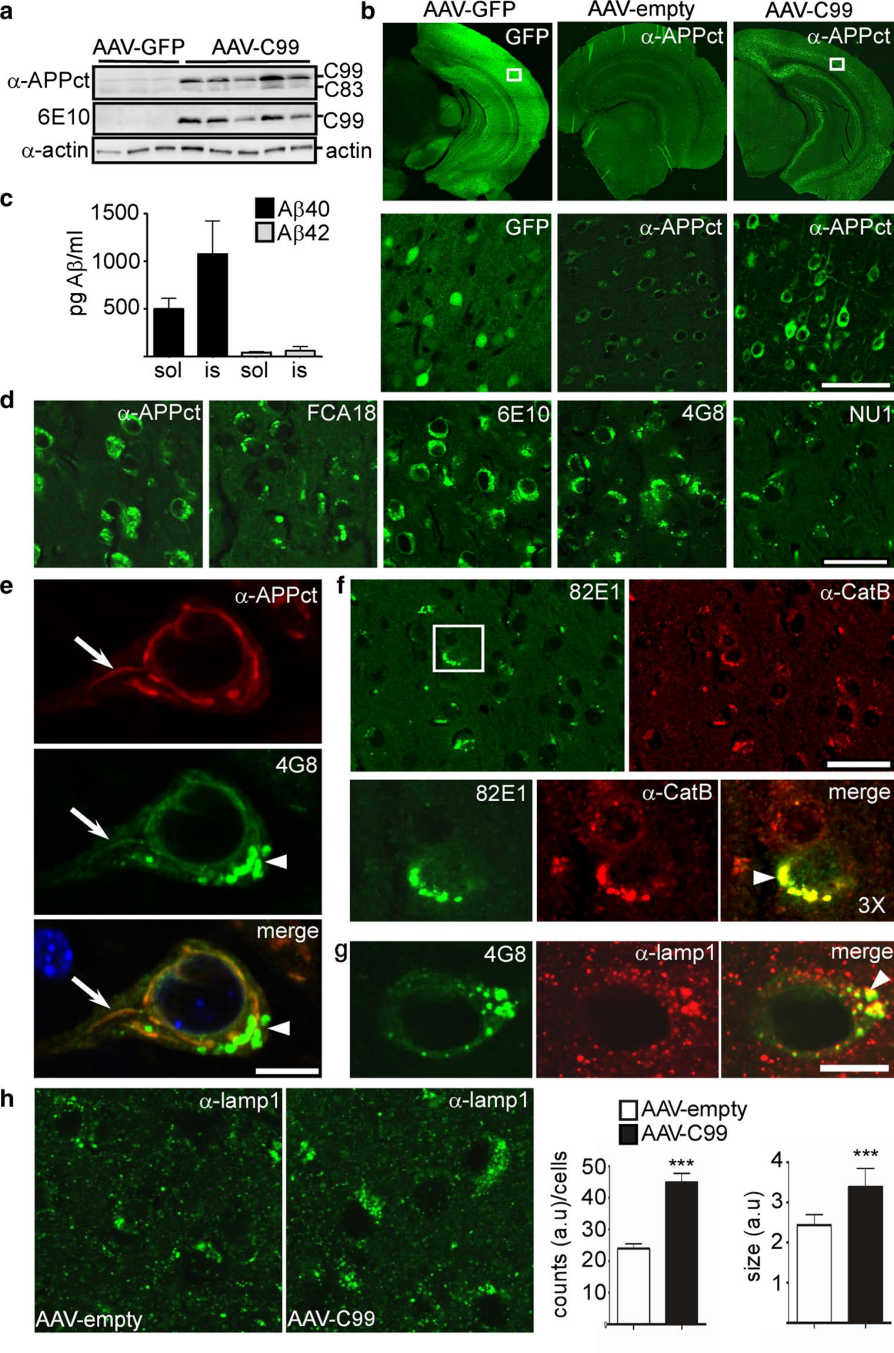
In vivo adeno-associated viral vector (AAV)-mediated expression of C99 in wild-type mice leads to the accumulation of aggregated C99 in enlarged cathepsin B-positive structures. 2-month-old AAV-empty, AAV-GFP or AAV-C99 were analyzed by western blot (**a**) or immunohistochemistry (**b**). **a** RIPA-soluble fractions from forebrain hemispheres were analyzed for APP-CTF expression using either α-APPct or 6E10. **b** GFP fluorescence was observed in AVV-GFP mice using a confocal microscope and C99 expression was visualized after immunohistochemical staining with α-APPct and using AAV-empty mice as a negative control. The *lower panel* shows higher magnification images at the level of the *boxed areas* highlighting the different subcellular labeling of GFP and C99. **c** *Bars* correspond to ELISA measurement of Aβ40 and Aβ42 in soluble (sol) and insoluble (is) fractions, respectively, and are represented as mean ± SEM *n* = 6 animals. **d** Images from immunohistochemical analysis of C99-associated expression in C99-AAV mice at the level of the subiculum using α-APPct, FCA18, 6E10, 4G8 or NU1. *Scale bar* 25 μm. **e** High-magnification images of co-immunolabeling with α-APPct and 4G8 illustrating the intraneuronal membrane-associated (*arrow*) and punctiform (*arrowhead*) stainings. *Scale bar* 5 μm. **f**, **g** Co-labeling of 82E1 and cathepsin B (**f**) or 4G8 and lamp1 (**g**) shows that C99 localizes to enlarged EAL-associated structures (*arrowhead* in merged images). *Scale bar* 25 and 5 μm, respectively. **h** Quantitative analysis of lamp1 structures in the subiculum of AAV-empty or AAV-C99 mice. Data are represented as the relative number per cell (counts (a.u)/cell) and average size of lamp1-positive puncta expressed as arbitrary units (a.u). Data are represented as mean ± SEM and are from two AAV-empty and two AAV-C99 mice, three brain slices for each animal and six images each slice. Images show representative images of lamp1 staining

In order to definitely demonstrate that C99 per se is sufficient to induce lysosomal pathology, we used an AAV-mediated strategy to express C99 under the transcriptional control of the neuron-specific synapsin I in wild-type animals. AAV-C99, AAV-GFP- or AAV-empty was injected into the ventricle of newborn mice and C99 expression was analyzed at 2 months post-AAV delivery. Western blot revealed a significant and specific increase of C99 in AAV-C99 injected brains (Fig. 6a). As compared to AAV-GFP mice, in which GFP was observed in many neurons and throughout the brain (Fig. 6b, left panels), immunohistochemical analysis of C99 in AAV-C99 mice using α-APPct, showed a more restricted expression that was particularly high in the hippocampus and neocortex (Fig. 6b, right panels). Within the neurons of these areas, the staining was intraneuronal and membrane-associated giving rise to a distinct immunostaining when compared to that of the cytoplasmic protein GFP (Fig. 6b). To discriminate between overexpressed C99 and endogenous APP and APP-CTFs that are also detected with the α-APPct antibody (recognizing both human and mouse APP, see supplementary table), we compared the immunostainings in AAV-C99 and AAV-empty mice. As expected, α-APPct detected membranous staining in many neurons and throughout the brain in AAV-empty mice; however, the intensity of this staining was much lower than that obtained in the AAV-C99 mice, indicating that the largest part of this staining corresponded to exogenously expressed C99. To determine the exact intraneuronal staining of C99, we performed immunocytochemical staining of C99 in cultured transfected cells that revealed that C99 was localized almost exclusively to the Golgi apparatus (Suppl. Figure 4a), whereas plasma membrane-associated C99 was also evident in few cells (Suppl. Figure 4b). As observed in SH-APPswe cells, the treatment with NH_4_Cl or D6 both led to the redistribution of APP-CTFs into EAL-associated structures and very little or no co-localization with GM130 was seen in these cells (Suppl. Figure 4c, d). In AAV-C99 brains, α-APPct did not detect EAL-associated staining, but interestingly when we used FCA18 or 82E1, the staining was punctiform and looked similar to that observed in the 3xTgAD mice. This labeling was present in the same brain regions labeled with α-APPct, but was localized to a much more restricted number of neurons and most strongly in the subiculum (Fig. 6d) and in some layers of the cortex. Interestingly, the other N-terminal-directed antibodies 6E10 and 4G8 recognized both the membrane-associated labeling and puncta (Fig. 6d, e—arrow and arrowhead, respectively) suggesting the presence of two distinct C99 species. Since, the lysosomal-associated staining required formic acid retrieval and since C99 has been shown to have a high tendency to self-aggregate and form amyloid-like fibrils in vitro [30, 60], we hypothesized that the C99 accumulating in lysosomes could correspond to aggregated or misfolded forms that would not be detected with C-terminal-directed antibodies because of aggregation-dependent structural or conformational changes of the C-terminal moiety. In agreement with this hypothesis, we found that the punctiform staining was also detected with the aggregate-specific antibody NU1 [23] (Fig. 6d). As observed in the 3xTgAD mice, these aggregates were localized to enlarged cathepsin B- and lamp1-positive structures (Fig. 6f, g) and the number and size of such enlarged structures was enhanced in the AAV-C99 mice, as compared to AAV-empty mice (Fig. 6h).

**Fig. 7:**
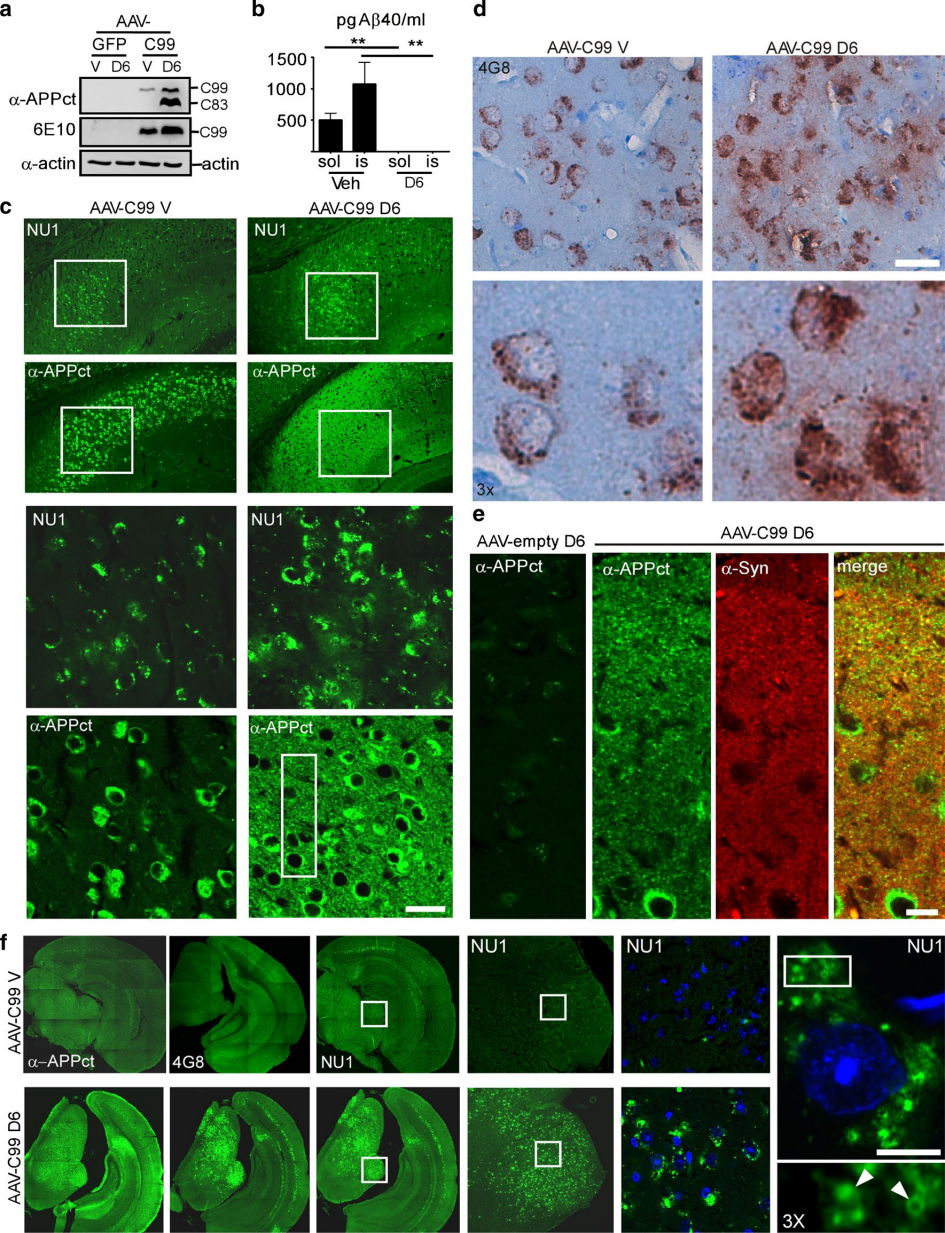
γ-Secretase inhibitor treatment of AAV-C99 expressing mice leads to increased accumulation of aggregated APP-CTFs within lysosomal-autophagic vesicles. AAV-C99 and AAV-empty injected mice were treated daily for 12 days with ELND006 (D6) or vehicle (V). **a** RIPA-soluble fractions of forebrain hemispheres were analyzed by western blot for APP-CTF expression using α-APPct or 6E10. **b** ELISA analysis of Aβ40 levels in RIPA-soluble- (sol) and formic acid retrieved (is) hemisphere fractions. Data are represented as mean ± SEM. Statistics were performed with the Mann–Whitney test by comparing soluble and insoluble fractions separately,**p < 0.01 (*n* = 6). **c** Images from immunofluorescence staining of brain slices from vehicle- (AAV-C99V) or D6-treated (AAV-C99 D6) AAV-C99 mice at the level of the subiculum using either NU1 or α-APPct. The *lower panels* correspond to high-magnification of the *boxed areas*. *Scale bar* is 125 and 20 μm, respectively. **d** Immunohistochemical staining with 4G8 of brain slices from AAV-C99V or AAV-C99 D6 mice using peroxidase/DAB development (brown staining). *Scale bar* is 20 μm. **e** Co-immunostaining of α-APPct and α-synaptophysin (α-Syn) revealed a high overlap within the subiculum of AAV-C99-D6 mice, but not in D6-treated AAV-empty mice (*left panel*). *Scale bar* is 5 μm. **f** Immunohistochemical staining of brain slices of AAV-C99 V (*upper panels*) or AAV-C99 D6 (lower panels) mice using α-APPct, 4G8 and NU1. *Middle* and *right panels* correspond to medium- and high-magnification images of NU1 labeling at the level of the *boxed areas*. Note the vesicular membrane-like staining of NU1 in *right panel* (*arrowheads*). Nuclei were stained with DAPI. *Scale bar* is 2.5 μm

**Fig. 8:**
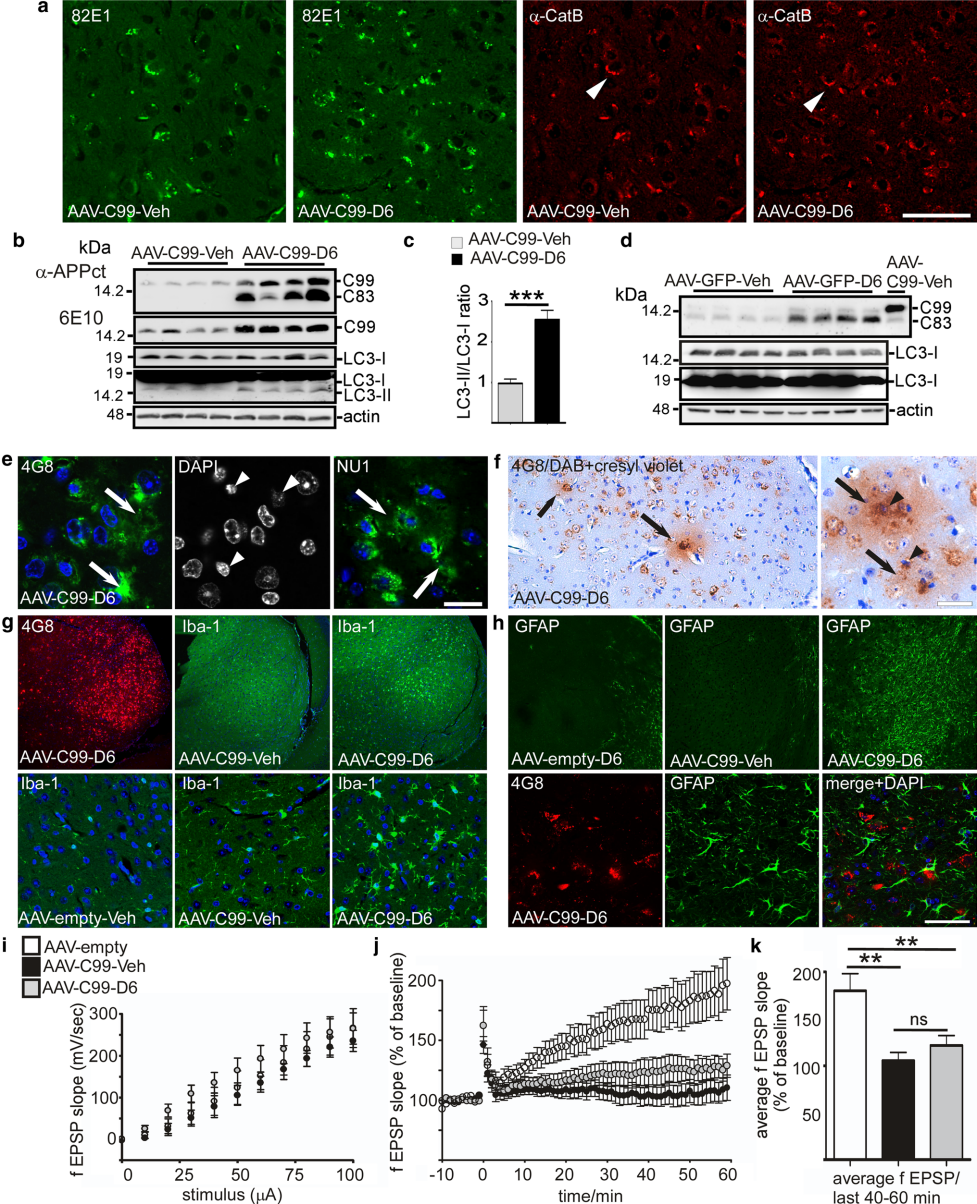
γ-Secretase inhibitor treatment of AAV-C99 expressing mice leads to exacerbated autophagic dysfunction, inflammatory responses and synaptic dysfunction. **a** Brain slices at the levels of the subiculum from vehicle- (Veh) or D6-treated AAV-C99 mice were immunostained with 82E1 and α-CatB. *Scale bar* 25 μm. **b**–**d** Western blot analysis of RIPA-soluble fractions from AAV-C99 injected mice (**b**, **c**) or AAV-GFP mice (**d**) treated with D6 or vehicle (Veh). Brains were analyzed for APP-CTF (using either α-APPct or 6E10) or LC3-I/LC3-II expression. *Bars* in **c** correspond to the quantitative analysis of LC3-II, expressed as the LC3-II/LC3-I ratio, and are relative to control (AAV-C99-Veh). Mann–Whitney test, *p* < 0.001, *n* = 8. *Right lane* corresponds to AAV-C99 mice. **e**, **f** Immunostaining with 4G8 or NU1 of D6-treated AAV-C99 brains using fluorescence (**e**) or peroxidase-DAB labeling (**f**). Note the extracellular staining (*white arrows* in **e** and *black arrows* in **f**) surrounding pycnotic nuclei visualized by DAPI (**e**, *white arrowheads*) or Cresyl violet (**f**, *black arrowheads*). *Scale bars* 50 or 10 μm, respectively. **g**, **h** Immunostaining with 4G8, Iba1 (**g**) or GFAP (**h**) reveals both microglial and astrocytic activation in D6-treated mice within brain regions expressing important APP-CTF levels. *Scale bar* 300 and 50 μm, respectively. **i** Basal synaptic transmission in AAV-empty, vehicle-treated AAV-C99 or D6-treated AAV-C99 mice (*n* = 2–3 slices per mouse from 6 to 7 mice per group. All values are mean ± SEM. **j** Field excitatory postsynaptic potential (fEPSP) slopes in subiculum (*n* = 2–3 slices per mouse from 6 to 7 mice per group). **k** Summary graph of LTP magnitudes calculated 40 to 60 min after high-frequency stimulation from graphs in (**j**) with statistical analysis (*p < 0.05; one-way ANOVA with the Tukey’s post hoc test). *Error bars* represent SEM

ELISA showed that C99-expressing mice do produce Aβ, corresponding mainly to Aβ40 but only very low levels of the aggregation-prone Aβ42 (Fig. 6c). Therefore, one could not exclude the possibility, that in this model, the punctiform staining could correspond to aggregated Aβ, instead of C99. Thus, to rule out this possibility, the C99-expressing mice were treated with D6. As described for 3xTgAD mice, γ-secretase inhibition enhanced the levels of C99 and C99-derived C83 [16] (Fig. 7a, but see also Fig. 8b) and strongly reduced Aβ levels (Fig. 7b). Immunohistochemical analysis using NU1 showed an increased rather than decreased punctiform immunostaining in D6-treated mice, clearly indicating that this staining should be accounted for C99 and not Aβ (Fig. 7c). Similar enhanced punctiform staining was observed with other N-terminal directed antibodies, 82E1 (Fig. 8a), FCA18, 6E10 (not shown) and 4G8 (Fig. 7d). In D6-treated mice, in areas expressing particular high punctiform staining, NU1 and 4G8 also gave rise to an extracellular diffuse staining surrounding the neurons (Fig. 7c, d, respectively), probably corresponding to the release of intraneuronal material from dying neurons (see also below). We also used α-APPct to analyze the effect of D6-treatment (Fig. 7c, e) and found that, within the subiculum, D6 also led to a strong APP-CTF associated staining localizing to synaptic regions (note the high overlap of α-APPct and the presynaptic marker synaptophysin in D6 treated AAV-C99 mice (Fig. 7e). The low intensity of α-APPct labeling in D6-treated AAV-empty mice showed that these synaptic localized APP-CTFs were mostly linked to viral-mediated C99. These observations confirmed the presence of two species of C99 (APP-CTFs), one non-aggregated and recognized by α-APPct and one aggregated and detected with N-terminal- and aggregate-specific antibodies. D6 treatment led to increased levels of both non-aggregated APP-CTFs localizing in presynaptic regions and of aggregated APP-CTFs accumulating in lysosomal-derived structures. It is noteworthy that similar D6-induced increases in both synaptophysin-positive APP-CTFs (Suppl. Figure 5a, d–e) and NU1-labeled aggregated APP-CTFs (Suppl. Figure 5b) was found in 3xTgAD mice, thus ruling out the possibility of artificial routing of C99 in the C99-expressing mice model. Moreover, western blot analysis confirmed the accumulation of C99 and C83 in both microsome- and synaptosome-enriched fractions of hippocampi from D6 treated 3xTgAD mice (suppl. Figure 5c).

Furthermore, and more surprisingly, in the C99-expressing mouse, D6 not only led to an enhanced staining in the cortex and subiculum, the two main regions expressing C99, but it also induced a strong immunostaining in various other brain regions, in which very low or no C99 staining was seen in vehicle-treated AAV-C99 mice (Fig. 7f), but in which GFP expression was high in AAV-GFP mice (Fig. 6b, left panels). Again, the labeling obtained with N-terminal antibodies was punctiform and in some regions also diffuse and extracellular. Taken together, these findings clearly demonstrated that this labeling should be ascribed to C99 and not to Aβ. Ηowever, they also suggested that these regions display either high γ-secretase activity or alternative D6-sensitive C99 degradation, explaining the absence of C99 labeling in vehicle-treated C99-expressing mice.

### D6 treatment not only leads to autophagic dysfunction but also to inflammatory responses and synaptic defects in C99-expressing mice, but not in wild-type mice

As described above, C99 accumulated in enlarged cathepsin B- and lamp1-positive structures and particularly within the subiculum in C99-expressing mice. In brains from D6-treated AAV-C99 mice, the number of neurons displaying such enlarged structures was increased rather than decreased (Fig. 8a). Furthermore, these brains displayed elevated levels of the autophagic marker LC3-II (Fig. 8b, c), while this was not the case in those from D6-treated AAV-GFP mice (Fig. 8d), again indicating that these lysosomal alterations were linked to C99 expression. Moreover, as described above, in some regions in which lysosomal-associated C99 was abundant, 4G8 also unraveled an important and diffuse extracellular staining likely corresponding to the release of intracellular material from dying neurons (Fig. 8e, f, but see also Fig. 7c, d). In agreement with necrotic cell death, nuclear staining with DAPI or Cresyl violet revealed the presence of many pycnotic nuclei (Fig. 8e, f). The same regions also displayed an increased number of both Iba-1-positive microglia (Fig. 8g) and GFAP-positive astrocytes (Fig. 8h). Iba-1 labeling was enhanced by the sole expression of C99 (compare AAV-C99 and AAV-empty) and further enhanced by D6 treatment, indicating concomitant C99 accumulation and inflammatory responses (Fig. 8g). Importantly, microglial and astrocytic activation (Fig. 8h) were not induced by D6 in AAV-empty mice. Finally, we assessed the effects of C99 expression on synaptic function by measuring long-term potentiation (LTP) at the CA1 pyramidal neurons to subicular neurons in AAV-empty or AAV-C99 vehicle or D6-treated mice (Fig. 8i–k). Whereas no differences were seen in basal synaptic transmission between AAV-empty, AAV-C99 vehicle or D6-treated mice (Fig. 8i), only the AAV-empty mice displayed a robust induction in LTP (Fig. 8j, k). D6 did not worsen LTP alterations, but this could be due to the drastic floor effect of C99 over-expression alone. However, the fact that D6 did not reverse synaptic dysfunction indicated that it was not caused by Aβ, but should be directly linked to C99.

## Discussion

Recently, we showed that the 3xTgAD mouse model develops an early, age-dependent intraneuronal accumulation of the β-secretase-derived βAPP fragment C99 [24]. In the present work, we demonstrate a link between this accumulating C99 and endosomal-autophagic-lysosomal (EAL) pathology. Firstly, the comparison of 2xTgAD (harboring wild-type PS1) and 3xTgAD mice (harboring PS1M146V) revealed that C99 accumulation was the consequence of impaired lysosomal-autophagic function rather than of altered γ-secretase processing and in agreement with this, C99 was found to accumulate within endolysosomal/autolysosomal structures in both of these mice. Moreover, our in vitro data and earlier work [1, 8, 57] demonstrated a major role of the lysosomal-autophagic pathway in C99 degradation. The 3xTgAD mice harbor PS1 and tau mutations, and the 2xTgAD mice harbor mutated tau, which possibly could have interfered with C99/APP trafficking and degradation, as described for some PS1 and tau mutants [25, 63]. Therefore, to determine whether EAL-associated C99 accumulation could be seen also in a model devoid of any mutated transgenes, we generated a viral-based C99-expressing transgenic model. Interestingly, in these mice immunohistochemical studies revealed the presence of two clearly distinct C99 species. One of it was intraneuronal membrane-associated and mainly localizing to the golgi apparatus. This C99 was recognized by α-APPct and representing the major part of C99. Another one was localized to EAL structures and restricted to a subgroup of the neurons. This C99 was aggregated or misfolded and recognized by both N-terminal-directed and aggregate-specific antibodies. Also in these mice, many of the C99-accumulating EAL vesicles were enlarged, again implying a link between C99 and EAL pathology. Taken together, these data ruled out the possibility that C99 aggregation and lysosomal localization was linked to artificial cellular trafficking caused by the expression of other mutated transgenes or to a putative lack of adequate targeting of exogenously expressed C99. We cannot exclude that the overexpression of these proteins itself could also have influenced degradation, but importantly elevated C99 levels have also been reported in human AD post-mortem brains [18, 22, 42], indicating that C99 accumulation is not specific to mouse AD models, but also do occur in human AD.

The EAL-associated C99 was recognized by the aggregate-specific antibody NU1 and localized to the membranes of these vesicles. We therefore hypothesized that this aggregated or misfolded C99 itself could also be a trigger of lysosomal/autophagic dysfunction. To validate this hypothesis, we took advantage of a pharmacological approach in which 3xTgAD or C99-expressing mice were treated with the γ-secretase inhibitor, ELND006 (D6) that enhances C99 levels [24]. Indeed, in both mouse models, D6 treatment increased EAL-associated C99 levels and led to exacerbated pathology: elevated levels of the autophagic markers LC3-II and p62, increased number of abnormal-sized C99-positive cathepsin B- and lamp1-structures and a higher density of non-digested autophagic vesicles highlighted by electron microscopy. Ultrastructural analysis also revealed the presence in neuropil of large autophagic vesicles filled with aberrant storage material likely corresponding to immature AVs, suggesting that, in addition to the effect on lysosomal proteolysis, γ-secretase inhibition also led to disturbed AV maturation and/or transport. Importantly, these effects of D6 were not observed in wild-type animals, strongly suggesting that they were dependent on APP-CTF accumulation. This was confirmed in an in vitro neuroblastoma model, since D6 led to autophagic impairment in APPswe overexpressing cells (SH-APPswe), but not in cells only expressing endogenous levels of APP (SH-mock).

Overall, the above set of data unveiled a pathological loop in which C99 accumulation was not only the result of altered autophagy, but could also be the cause of it. The observations that aggregated C99 accumulated within the membranes of the EAL vesicles suggested that it likely interfered directly with EAL function by perturbating membrane integrity and inhibiting normal lysosomal proteolysis. Possibly exacerbated C99 accumulation could also lead to the leakage of catabolic contents, lysosomal enzymes and acidity into the cytoplasm and to resulting neuronal cell death [17, 28]. Indeed, both electron microscopy and immunohistochemical analysis revealed clear signs of neuronal necrosis and release of aggregated C99 (and C99-derived C83) to extracellular spaces. Strikingly, recent papers described similar deleterious effects of EAL-associated Aβ42 in a transgenic Aβ42-expressing drosophila model [26–28], suggesting that whatever the nature of the aggregates, their accumulation within EAL-vesicle membranes could disturb lysosomal-autophagic function. In the D6-treated AAV-C99 mice, C99 accumulation was also associated with strong inflammatory responses including both microglial activation and astrogliosis. These findings showed that the pathology observed in the C99 mice resembled that seen in lysosomal storage disorders, in which a primary defect of lysosomal function induces both autophagic impairment and the activation of the immune system as a response to the accumulation of storage material and/or dying neurons [44]. Interestingly, we also observed that C99 expression strongly affected synaptic function. Long-term potentiation recordings in the subiculum of the AAV-injected mice, showed an almost complete absence of LTP in the subiculum of C99-expressing mice. D6 did not reverse this effect clearly indicating that it was linked to C99 expression itself and not to Aβ.

Altogether, our data showed a direct and clearly Aβ-independent detrimental effect of C99 and provide new molecular explanations for earlier mechanistically unsolved studies describing APP-CTF-linked toxicity. For instance, it was reported that the conditional knock-out of presenilin1 in mice led to a progressive development of synaptic and cognitive alterations that were temporally correlated with APP-CTF accumulation [48]. Moreover, the treatment with γ-secretase inhibitors was found to induce both long-term deficits and APP-CTF accumulation in mice [31, 52] and to accelerate cognitive decline in human [15]. Consistent with these findings, the work from the group of Dr d’Adamio showed that the inhibition of β-secretase but not of γ-secretase led to reduced synaptic/memory defects in a mouse model of familial dementia [53]. Finally, the transgenic overexpression of BACE-1 in mice was observed to induce a pathogenic pathway involving the accumulation of APP-CTFs but not of Aβ [46]. Recent work from the group of Dr Nixon proposed a direct link between C99 accumulation and endocytic abnormalities in fibroblasts derived from Down’s syndrome patients [20, 22], but these studies did not investigate the downstream effects of C99 accumulation on lysosomal and autophagic function.

This work is the first to demonstrate the presence of two structurally and immunologically distinguishable C99 species, one corresponding to non-aggregated and intraneuronal membrane-bound C99 and one to aggregated or misfolded C99 localized to EAL-vesicle membranes. The pharmacological inhibition of γ-secretase led to drastic increases in both non-aggregated C99 accumulating within synaptic regions, and in aggregated EAL-associated C99. These findings therefore reconciliate previous works demonstrating different localizations of APP-CTFs. Whereas some studies reported presynaptic APP-CTF accumulation after γ-secretase inhibition [5, 31] or in presenilin knock-out mice [48], others found lysosomal APP-CTF accumulation in presenilin-invalidated cultured cells [14].

Altogether, our findings add a new toxic trigger to the amyloid cascade, which could underlie some of the AD-related hallmarks occurring especially in the early stages of the disease. In the 3xTgAD mouse, the intraneuronal C99 accumulation occurs earlier and is more important than that of Aβ and in the AAV-C99 mice, the aggregation-prone Aβ42 is only produced in very low amounts, compared to that of Aβ40. These findings might explain the major contribution of C99 to the observed phenotypes in our models, but of course we do not exclude the contribution of Aβ to the pathology. Finally, our data also show a region-specific accumulation of C99, indicating differences in proteolytic processing and degradative mechanisms within neuronal subtypes. These differences could explain the accumulation of distinct catabolites in specific neuronal populations and underlie mechanisms contributing to neuronal susceptibility to AD-related pathology. Taken together, our study demonstrates that C99 can participate to lysosomal pathology in the absence of Aβ.

## Electronic supplementary material

Below is the link to the electronic supplementary material.

2xTgAD and 3xTgAD mice display early and progressive C99 accumulation. **a** Immunohistochemical staining with FCA18 on brain slices of 6, 12 or 22 month-old 2xTgAD (2AD) and 3xTgAD (3AD) mice at the level of the subiculum. **b** Aβ42 immunostaining in 22 month-old 2AD and 3AD mice. Scale bar: 100 μm. **c** Western blot analysis of C83 and C99 expression in 3- or 12 month-old non-transgenic (nonTg), 2AD and 3AD mice. **d** Bars correspond to the quantification of C83 and C99 levels in 12-month-old 2AD (grey bars, n=11) and 3AD (black bars, n=11) mice. **e** Bars indicate ELISA analysis of Aβ40 and Aβ42 levels in acid formic extracted hippocampi of 12 month old 2AD (grey bars, n=5) and 3AD (black bars, n=5) mice. Animals were all males. Data are represented as mean ± s.e.m, and statistics were performed using the Mann Whithey test, **p<0.01 (TIFF 3838 kb)

Ultrastructural analysis of vehicle or γ-secretase inhibitor treated non transgenic or 3xTgAD mice. The Figure shows electron microphotographs of neuronal somas and neuropil from a vehicle-treated nonTg mouse (nonTg-CT) (**a**, **c**), a D6-treated nonTg mouse (nonTg-D6) (**b**, **d**) or D6-treated 3xTgAD mouse (AD-D6) (**e-j**). Both vehicle and D6-treated nonTg mice presented few autophagic vesicles, and displayed normal appearing neuropil with a high number of synaptic contacts (yellow arrows) and normal-appearing mitochondria (black arrows). In contrast, D6-treated 3xTgAD mouse brains presented many typical dense giant autolysosomes (red arrows) and multilamellar bodies (blue ML) as well as large vesicles filled with heterogenous material (red arrowheads). BV corresponds to a brain vessels, blue star to electron-lucent areas and blue N to the nucleus. Scale bar is 5 μm in **a**, **b**, **f** and **j**, 2 μm in **c**, **d**, **g** and **h** and 10 μm in **e** (TIFF 17479 kb)

γ-secretase inhibitor treatment in 2xTgAD and 3xTgAD mice leads to identical increases in APP-CTF levels and intraneuronal punctiform staining. 5 month-old 2xTgAD (2AD) and 3xTgAD (3AD) mice were treated during 12 days with ELND006 (30 mg/kg) and analyzed for APP-CTF levels by western blot using α-APPct (**a-b**) or for Aβ42 levels in acid formic retrieved fractions by ELISA (**c**). Bars in **b** correspond to the quantitative analysis of C99, C83 and AICD obtained in **a,** and are relative to the levels expressed in vehicle treated 2AD mice (2ADveh). Data are represented as mean ± s.e.m, as determined by ANOVA one-way Tukey’s post hoc test, ***p<0.001. n=6 animals for each genotype and each treatment. No statistical analysis was performed for AICD, which was not detected on all gels. **d** C99 expression was visualized by immunohistochemistry using FCA18. Left panel corresponds to low-magnification images of D6-treated 2AD and 3AD mice, at the level of the subiculum. Right panels show higher magnification images of vehicle or D6-treated 2AD and 3AD mice. Blue staining corresponds to DAPI. Scale bar is 100 μm and 25 μm, respectively (TIFF 4006 kb)

C99 expressed in COS-7 cells. Co-staining of C99 with α-APPct and the cis-golgi marker GM130 showed that C99 in most cells was localized exclusively within the golgi apparatus (**a**). However, some cells also displayed clear plasma-membrane staining of C99 (**b**). In cells treated with NH_4_Cl or D6, C99 was relocalized to EAL-associated structures and no or very few co-staining was found with the cis-golgi marker GM130 (**c-d**). Scale bar = 20 μm (TIFF 3520 kb)

In 3xTgAD mice, the γ-secretase inhibitor leads to increased levels of APP-CTFs within both synaptic regions and EAL compartments. **a**, Brain slices at the levels of the subiculum from vehicle- (AD-CT) or D6-treated (AD-D6) 3xTgAD mice were immunostained with α-APPct. The images at the right hand correspond to high-magnification images of the boxed ares. Scale bar is 125 μm and 20 μm, respectively. **b**, D6-treated brain sections were co-immunostained with NU1 and FCA18. Note the perfect overlap in merged image. Scale bar is 125 μm and 20 μm, respectively. **c** Western blot analysis of βAPP and APP-CTF expressions in microsomal- (M) or synaptosomal-enriched (S) fractions from hippocampi of AD-CT or AD-D6 mice. Note that C99 and C83 accumulate in both fractions in D6-treated mice. **d**-**e,** Images from brain slices at the levels of the subiculum from AD-CT or AD-D6 mice. **e** slices were co-immunostained with α-APPct and α-synaptophysin. See the high overlap of staining in AD-D6 mice (merge images). Scale bar is 250 μm and 50 μm, respectively (TIFF 15670 kb)

Antibodies used in this study (TIFF 611 kb)
