# Supplementary figures and images for: Intraneuronal aggregation of the β-CTF fragment of APP (C99) induces Aβ-independent lysosomal-autophagic pathology
Source: Acta Neuropathol. 2016 Apr 30;132(2):257–76. doi: 10.1007/s00401-016-1577-6 (PMC4947121; doi:10.1007/s00401-016-1577-6)

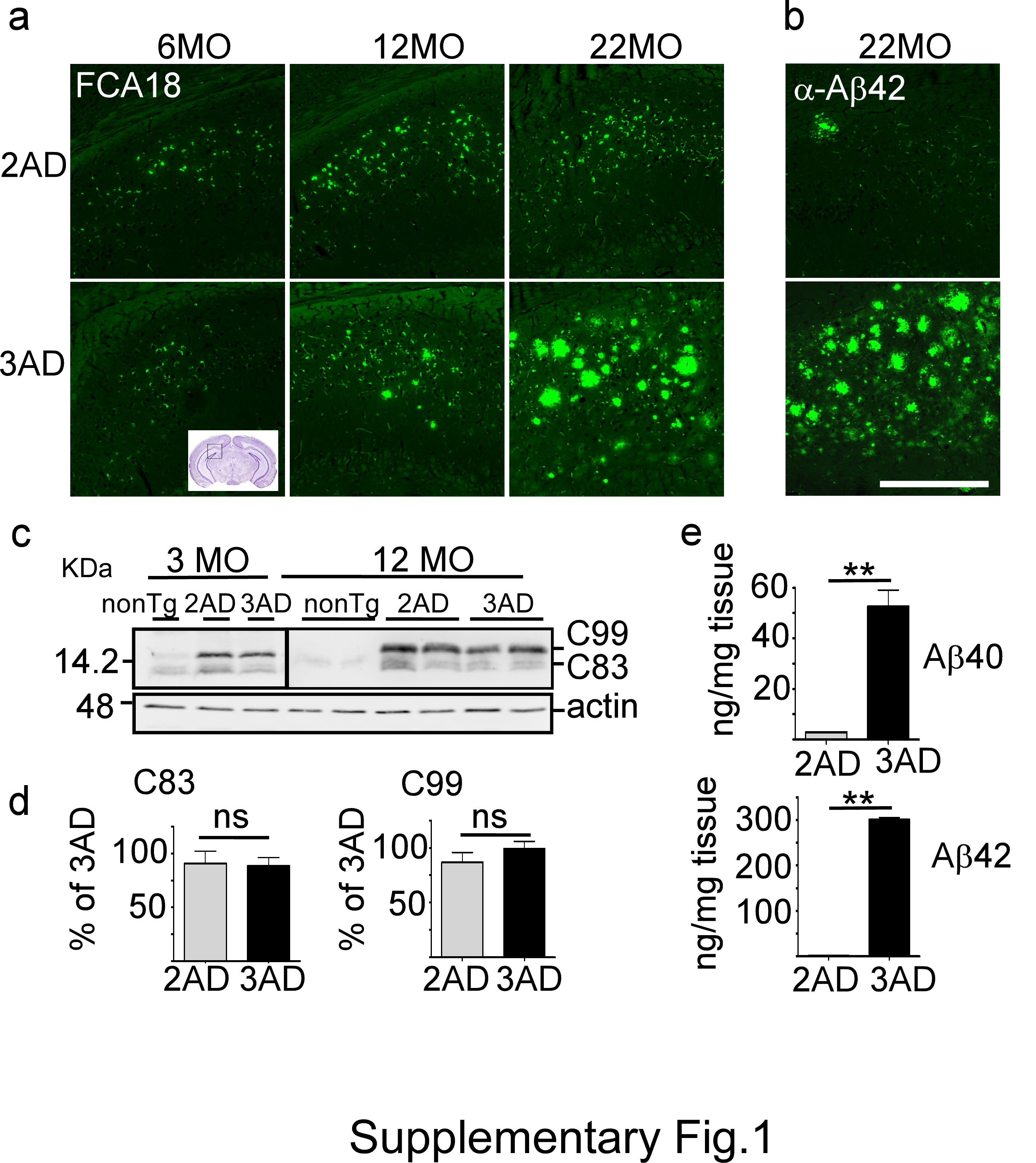

Supplement: Supplementary file 1 — 2xTgAD and 3xTgAD mice display early and progressive C99 accumulation. a Immunohistochemical staining with FCA18 on brain slices of 6, 12 or 22 month-old 2xTgAD (2AD) and 3xTgAD (3AD) mice at the level of the subiculum. b Aβ42 immunostaining in 22 month-old 2AD and 3AD mice. Scale bar: 100 μm. c Western blot analysis of C83 and C99 expression in 3- or 12 month-old non-transgenic (nonTg), 2AD and 3AD mice. d Bars correspond to the quantification of C83 and C99 levels in 12-month-old 2AD (grey bars, n=11) and 3AD (black bars, n=11) mice. e Bars indicate ELISA analysis of Aβ40 and Aβ42 levels in acid formic extracted hippocampi of 12 month old 2AD (grey bars, n=5) and 3AD (black bars, n=5) mice. Animals were all males. Data are represented as mean ± s.e.m, and statistics were performed using the Mann Whithey test, **p<0.01 (TIFF 3838 kb) [file 401_2016_1577_MOESM1_ESM.tif]

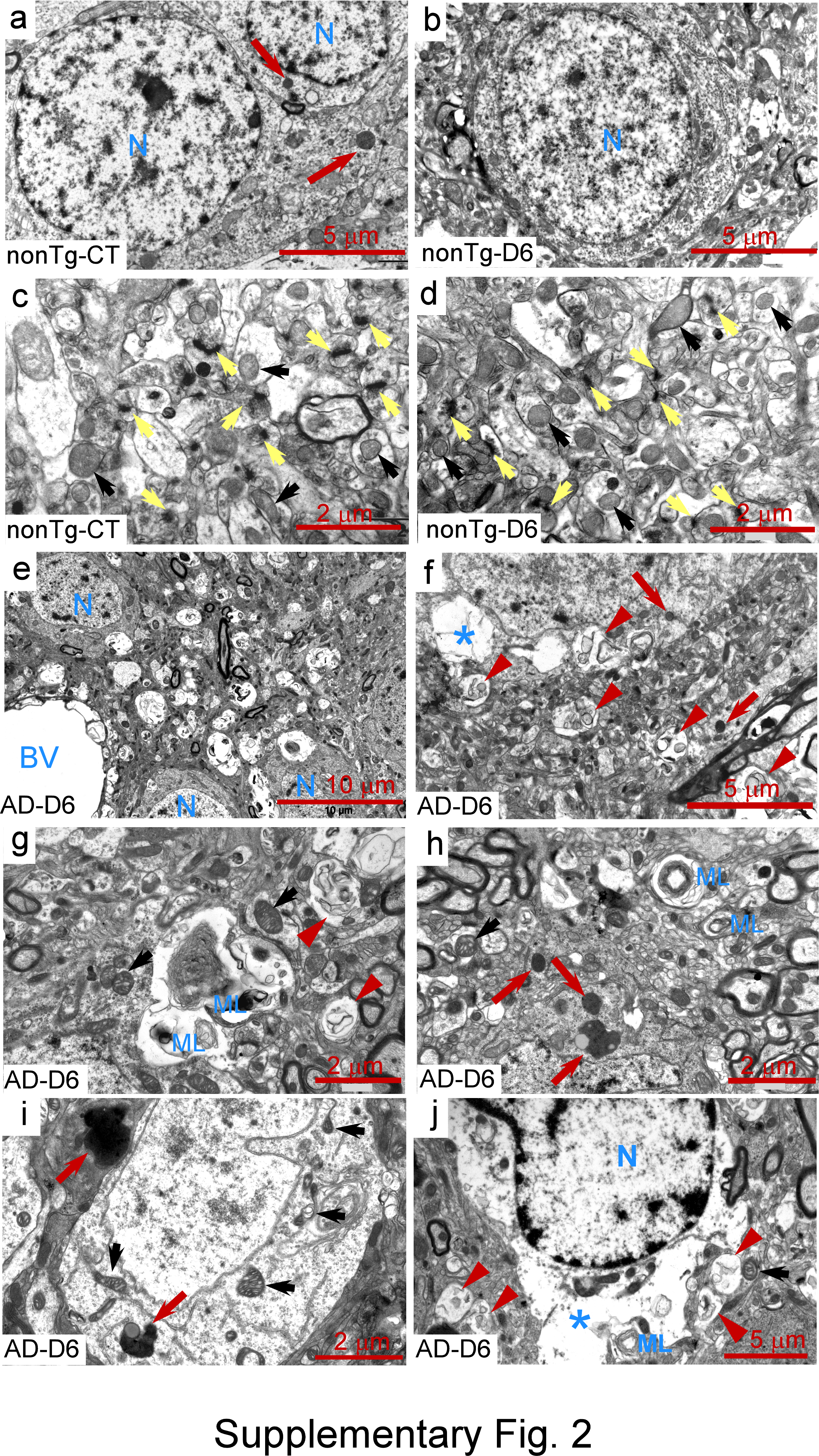

Supplement: Supplementary file 2 — Ultrastructural analysis of vehicle or γ-secretase inhibitor treated non transgenic or 3xTgAD mice. The Figure shows electron microphotographs of neuronal somas and neuropil from a vehicle-treated nonTg mouse (nonTg-CT) (a, c), a D6-treated nonTg mouse (nonTg-D6) (b, d) or D6-treated 3xTgAD mouse (AD-D6) (e-j). Both vehicle and D6-treated nonTg mice presented few autophagic vesicles, and displayed normal appearing neuropil with a high number of synaptic contacts (yellow arrows) and normal-appearing mitochondria (black arrows). In contrast, D6-treated 3xTgAD mouse brains presented many typical dense giant autolysosomes (red arrows) and multilamellar bodies (blue ML) as well as large vesicles filled with heterogenous material (red arrowheads). BV corresponds to a brain vessels, blue star to electron-lucent areas and blue N to the nucleus. Scale bar is 5 μm in a, b, f and j, 2 μm in c, d, g and h and 10 μm in e (TIFF 17479 kb) [file 401_2016_1577_MOESM2_ESM.tif]

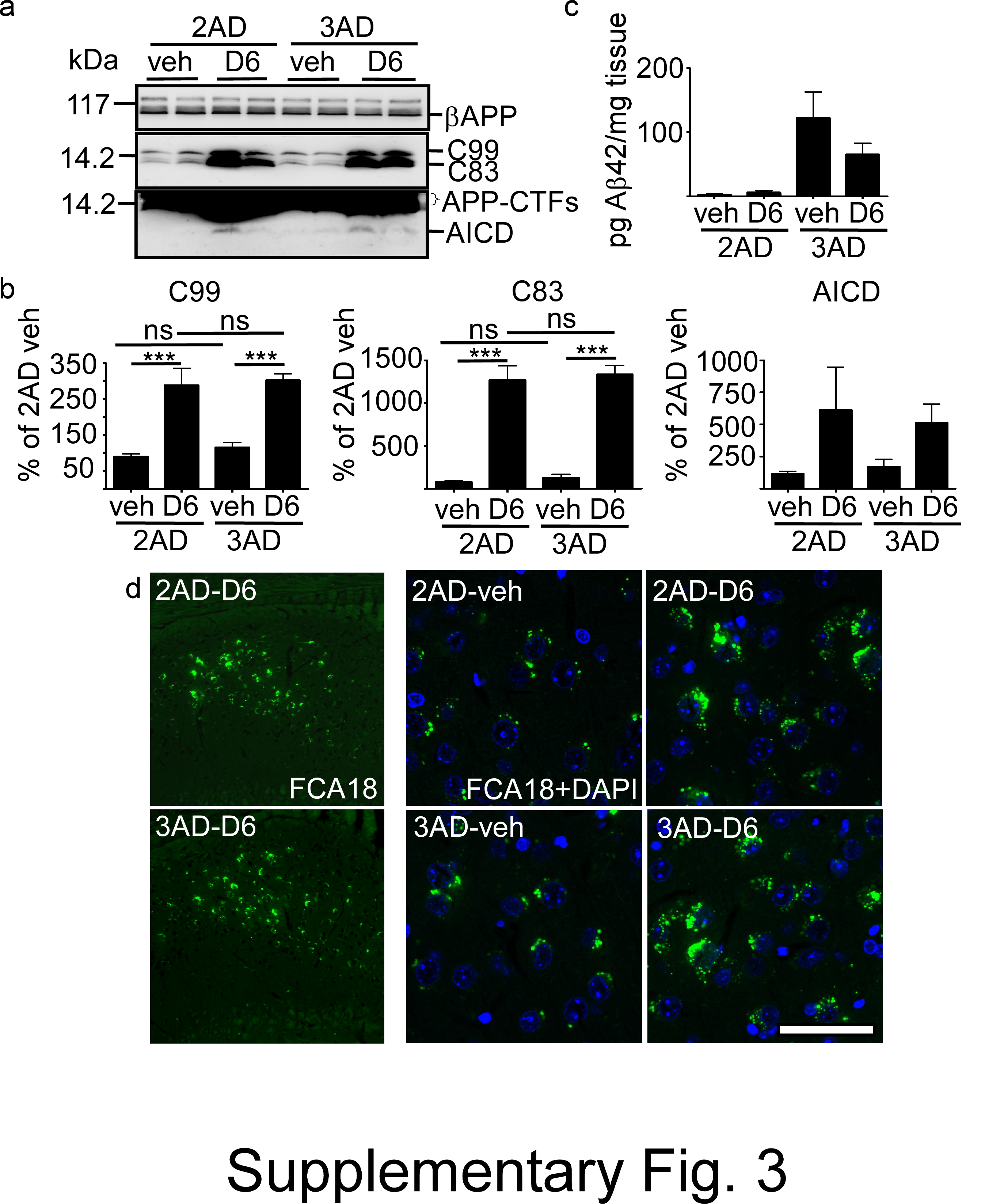

Supplement: Supplementary file 3 — γ-secretase inhibitor treatment in 2xTgAD and 3xTgAD mice leads to identical increases in APP-CTF levels and intraneuronal punctiform staining. 5 month-old 2xTgAD (2AD) and 3xTgAD (3AD) mice were treated during 12 days with ELND006 (30 mg/kg) and analyzed for APP-CTF levels by western blot using α-APPct (a-b) or for Aβ42 levels in acid formic retrieved fractions by ELISA (c). Bars in b correspond to the quantitative analysis of C99, C83 and AICD obtained in a, and are relative to the levels expressed in vehicle treated 2AD mice (2ADveh). Data are represented as mean ± s.e.m, as determined by ANOVA one-way Tukey’s post hoc test, ***p<0.001. n=6 animals for each genotype and each treatment. No statistical analysis was performed for AICD, which was not detected on all gels. d C99 expression was visualized by immunohistochemistry using FCA18. Left panel corresponds to low-magnification images of D6-treated 2AD and 3AD mice, at the level of the subiculum. Right panels show higher magnification images of vehicle or D6-treated 2AD and 3AD mice. Blue staining corresponds to DAPI. Scale bar is 100 μm and 25 μm, respectively (TIFF 4006 kb) [file 401_2016_1577_MOESM3_ESM.tif]

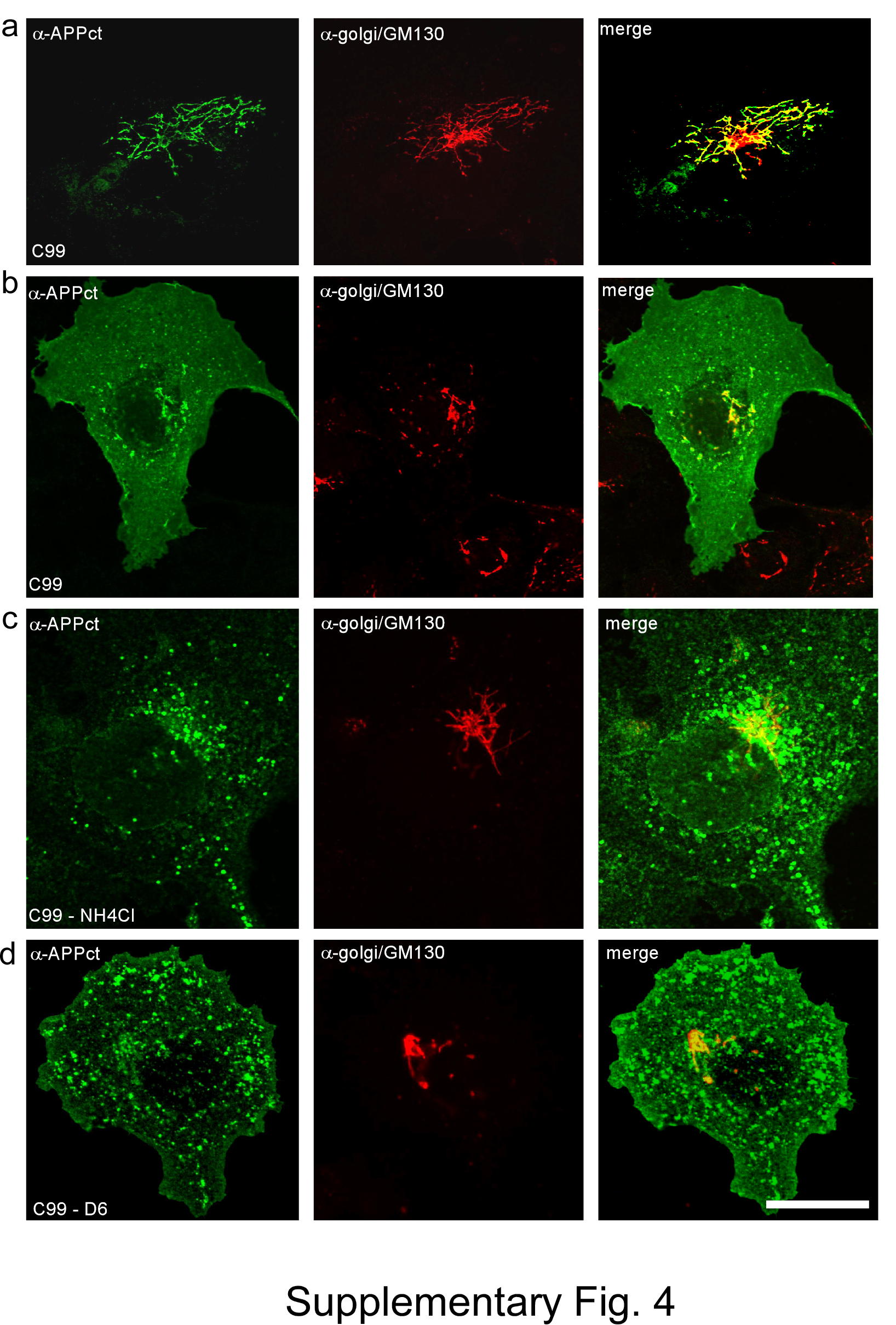

Supplement: Supplementary file 4 — C99 expressed in COS-7 cells. Co-staining of C99 with α-APPct and the cis-golgi marker GM130 showed that C99 in most cells was localized exclusively within the golgi apparatus (a). However, some cells also displayed clear plasma-membrane staining of C99 (b). In cells treated with NH4Cl or D6, C99 was relocalized to EAL-associated structures and no or very few co-staining was found with the cis-golgi marker GM130 (c-d). Scale bar = 20 μm (TIFF 3520 kb) [file 401_2016_1577_MOESM4_ESM.tif]

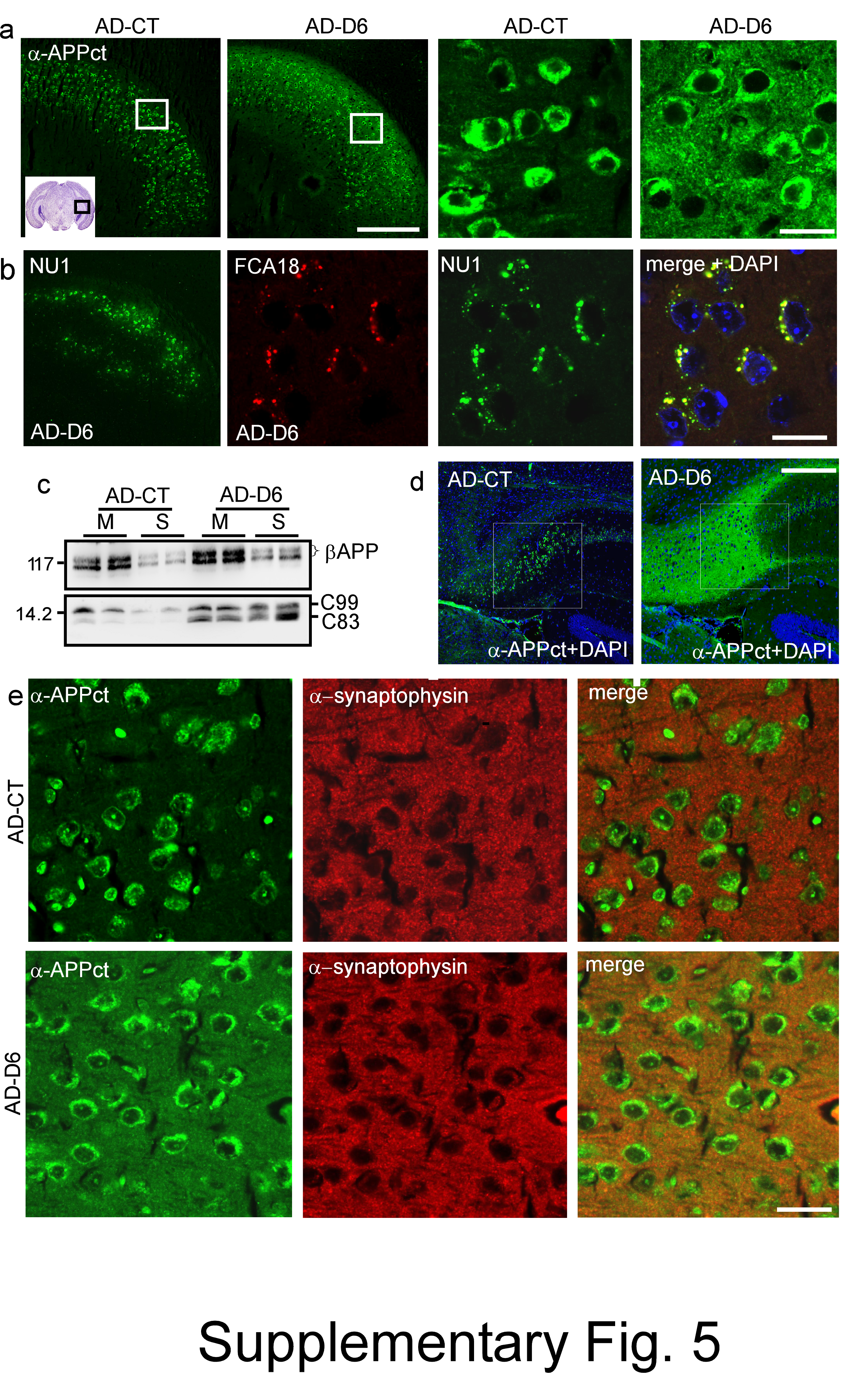

Supplement: Supplementary file 5 — In 3xTgAD mice, the γ-secretase inhibitor leads to increased levels of APP-CTFs within both synaptic regions and EAL compartments. a, Brain slices at the levels of the subiculum from vehicle- (AD-CT) or D6-treated (AD-D6) 3xTgAD mice were immunostained with α-APPct. The images at the right hand correspond to high-magnification images of the boxed ares. Scale bar is 125 μm and 20 μm, respectively. b, D6-treated brain sections were co-immunostained with NU1 and FCA18. Note the perfect overlap in merged image. Scale bar is 125 μm and 20 μm, respectively. c Western blot analysis of βAPP and APP-CTF expressions in microsomal- (M) or synaptosomal-enriched (S) fractions from hippocampi of AD-CT or AD-D6 mice. Note that C99 and C83 accumulate in both fractions in D6-treated mice. d-e, Images from brain slices at the levels of the subiculum from AD-CT or AD-D6 mice. e slices were co-immunostained with α-APPct and α-synaptophysin. See the high overlap of staining in AD-D6 mice (merge images). Scale bar is 250 μm and 50 μm, respectively (TIFF 15670 kb) [file 401_2016_1577_MOESM5_ESM.tif]

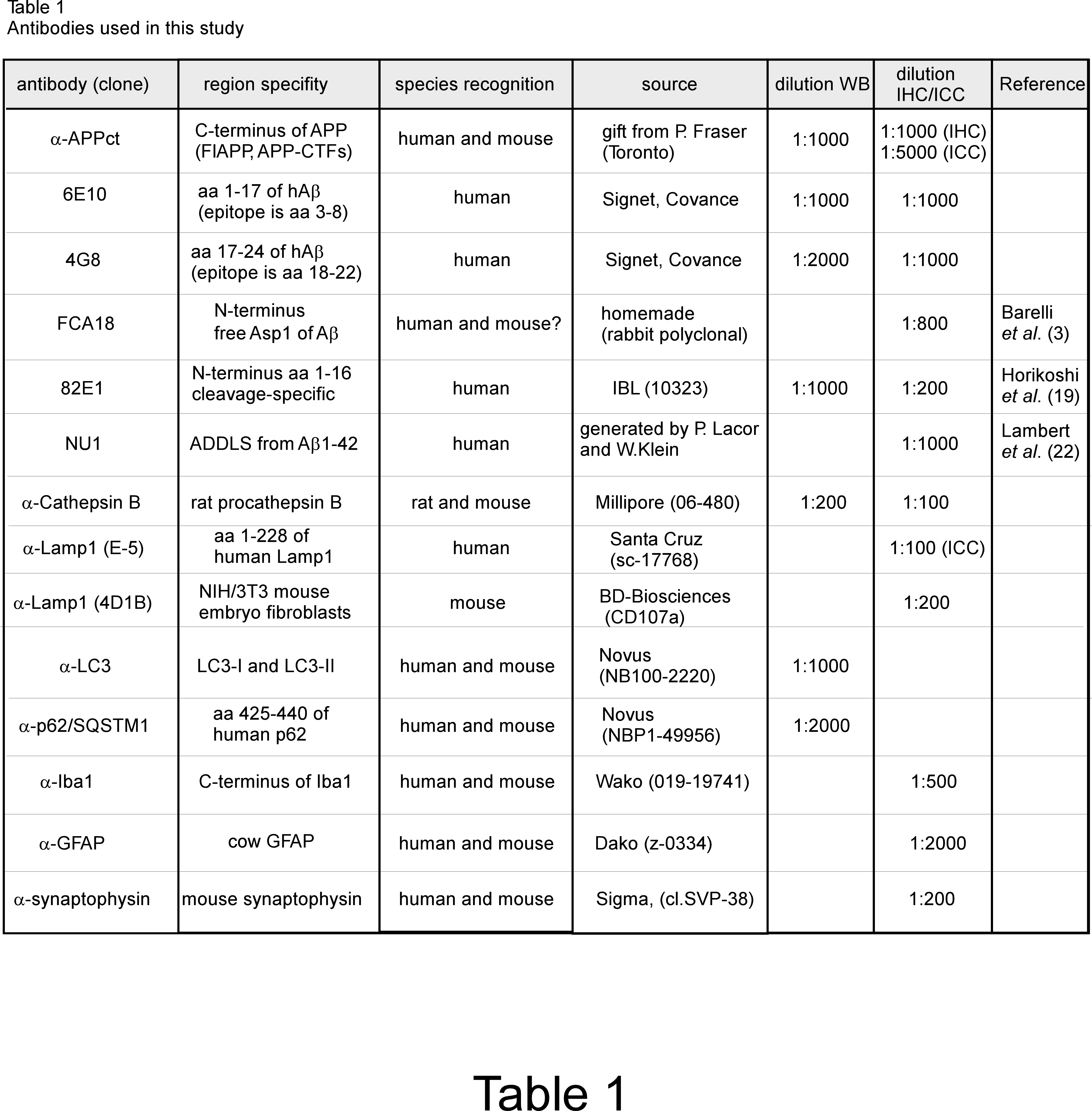

Supplement: Supplementary file 6 — Antibodies used in this study (TIFF 611 kb) [file 401_2016_1577_MOESM6_ESM.tif]
